# Synthetic liver fibrotic niche extracts achieve *in vitro* hepatoblasts phenotype enhancement and expansion

**DOI:** 10.1016/j.isci.2021.103303

**Published:** 2021-10-19

**Authors:** Yuying Zhang, Anqi Guo, Cheng Lyu, Ran Bi, Zhaozhao Wu, Wenjing Li, Peng Zhao, Yudi Niu, Jie Na, Jianzhong Jeff Xi, Yanan Du

**Affiliations:** 1 Department of Biomedical Engineering, School of Medicine, Tsinghua-Peking Center for Life Sciences, MOE Key Laboratory of Bioorganic Phosphorus Chemistry and Chemical Biology, Tsinghua University, Beijing 100084, China; 2 School of Life Sciences, Tsinghua University, Beijing 100084, China; 3 Center for Stem Cell Biology and Regenerative Medicine, School of Medicine, Tsinghua University, Beijing 100084, China; 4 State Key Laboratory of Natural and Biomimetic Drugs, Institute of Molecular Medicine, Department of Biomedical Engineering, College of Engineering, Peking University, Beijing 100871, China

**Keywords:** Tissue engineering, Biomimetics, Stem cells research

## Abstract

It is still a challenge for synthesizing ‘cellular niche-mimics’ *in vitro* with satisfactory reproducibility and fidelity to recreate the natural niche components (e.g., extracellular matrices and soluble factors) for stem cell cultivation. Inspired by the massive amplification of hepatic progenitor cells during liver fibrosis *in vivo*, here we optimized the *in vitro* liver fibrotic niches and subsequently harvested their bioactive ingredients as niche extracts (NEs). The fibrosis-relevant NE marginally outperformed Matrigel for phenotype maintenance of human embryonic stem cell (hESC)-derived hepatoblasts (HBs) and recapitulation of the pathological angiogenesis of hESC-derived endothelial cells both in 2D culture and 3D liver organoids. Finally, defined NE components (i.e., collagen III, IV, IL-17, IL-18 and M-CSF) were resolved by the quantitative proteomics which exhibited advantage over Matrigel for multi-passaged HB expansion. The pathology-relevant and tissue-specific NEs provide innovative and generalizable strategies for the discovery of optimal cellular niche and bioactive niche compositions.

## Highlights

• Fibrotic niches were constructed by 3 hepatic cell lines plus 4 profibrotic factors
• NE was produced by enzymatic digestion using pepsin and DNase
• Collagen III, IV, IL-17, IL-18, and M-CSF resolved from NE promoted HBs expansion

## Graphical abstract

**Figure:**
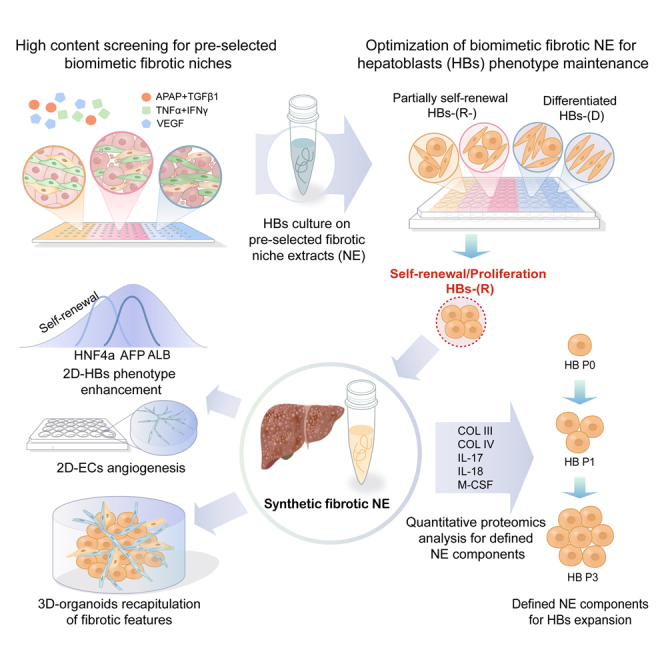

## Introduction

Functions and the fate of tissue-specific stem cells are highly dependent on their residing niche *in vivo*, which consists of diverse cell types, intricately distributed extracellular matrix (ECM), soluble factors and environmental factors (e.g., hypoxia and physical factors) (Lane et al., 2014). Due to the complexity of natural niches, it is still a challenge to *de novo* synthesize and recreate the biomimetic stem cell niche *in vitro*, which are favored for long-term phenotype maintenance and expansion of stem cells. Current strategies for obtaining ECM and bioactive factor composite *in vitro* that mimics the natural tissue-specific niche mainly include natural tissue decellularization (Tian et al., 2018; Wang et al., 2011) and ingredient harvesting from *in vitro-*cultured cells (Prewitz et al., 2013). For example, Wang et al. developed a four-step perfusion decellularization method for the preparation of decellularized rat liver ECM, which was able to remove cells while preserving the histological feature, the vascular structure, most of the collagen-related matrix components, and the physiological levels of matrix-bound factors (Wang et al., 2011). Human hepatic progenitor cells seeded on the decellularized liver ECM differentiated into mature and functional parenchymal cells within 1 week which could stabilize the mature phenotype for over 8 weeks (Wang et al., 2011). Tian et al. obtained the rat lung and liver decellularized ECMs which were further homogenized into stable matrix solution for coating of the cell culture plates (Tian et al., 2018). They found that the rectal cancer cells spontaneously formed 3D cell colonies on the lung and liver decellularized ECMs, which resembled the *in vivo* metastatic matrices better in comparison to commercial Matrigel and pure type I collagen (Tian et al., 2018). The decellularized tissue matrices could retain natural tissue-specific niche components and 3D architectures to maintain cellular phenotypes and functions (Miyauchi et al., 2017). However, shortage of human tissue supply, poor reproducibility due to batch-to-batch variations, and the difficulties to obtain decellularized tissues with different physiological states restricted their wide applications.

Meanwhile, ECM components secreted by *in vitro* primed mesenchymal stem cells (MSC) have been utilized as synthetic niche to promote the proliferation and maintain the phenotype of CD34^+^ hematopoietic stem cells (Prewitz et al., 2013). It should be noted that bioactive ingredients harvested from one type of primary cell could not recreate the interactions among multiple cell types in the natural hematopoietic stem cell niche (Prewitz et al., 2013). Furthermore, the primary cells, in particular of human origin, also suffer from batch-to-batch variation and limited supplies. Thus, current methods of constructing bioactive ECM mimetics based on tissue decellularization and cell culture extraction cannot satisfy the cultivation needs of tissue-specific stem cells *in vitro* (Lane et al., 2014).

Specifically, phenotype maintenance and expansion of liver progenitor cell types-hepatoblasts (HBs) *in vitro* could provide high-quality cell sources for regenerative therapy, liver tissue engineering, and establishment of *in vitro* liver pathophysiological models. Matrigel, specific ECM components (e.g., laminin-111) as well as a combination of growth factors (e.g., HGF and EGF) and small molecules (e.g., Wnt agonists and TGF-β inhibitors) have been reported to support HBs amplification *in vitro* (Lv et al., 2015; Takayama et al., 2013). However, the compositions of those matrices and additives were highly varied from the natural niche that HBs inhabit. Actually, amplification of liver progenitor cells residing in the periportal region was a hallmark in the mild stage of liver fibrosis to make up for the loss of hepatocytes during chronic liver injury (Font-Burgada et al., 2015; Williams et al., 2014). The mild stage liver fibrotic niche is constituted by abnormal ECM (Iwaisako et al., 2012), complex soluble factors, and multiple resident cell types [e.g., damaged or senescent hepatocytes (Kaur et al., 2015), activated hepatic stellate cells (HSCs) (Iwaisako et al., 2012), angiogenic liver sinusoidal endothelial cells (LSECs) and activated Kupffer cells]. Inspired by the HBs amplification in the fibrotic liver, we hypothesized that bioactive ingredients derived from biomimetic liver fibrotic niche would enhance phenotype maintenance during the multi-passaged expansion of HBs *in vitro*.

Here, we tested our hypothesis by first conducting high-content screening for biomimetic fibrotic niches, which were constructed on 3D microwell chips with combinations of 3 major cell lines corresponding to hepatocytes, HSCs and LSECs plus 4 pro-fibrotic factors. To preserve the pathological characteristics of the niche, a unique niche processing strategy that only removed the nucleus while retaining all the bioactive ingredients was performed to obtain the niche extracts (NEs) (Tian et al., 2018). Based on the analyzed correlations between fibrotic niche features and their regulatory effect on HBs, we optimized the biomimetic fibrotic niches and synthesized the resulting NEs, which could promote HBs’ phenotype maintenance both in 2D culture and 3D organoids configurations. Finally, defined NE compositions were resolved by the quantitative proteomics which outperformed Matrigel for HBs’ expansion *in vitro*.

The biomimetic fibrotic NEs and their defined compositions would satisfy the need of developing ECM-like matrices for large-scale expansion of HBs as high-quality stem cell supply. The concept of synthetic biomimetic niche and NE could also be applied to optimize *in vitro* expansion conditions for other tissue-specific stem cells with wider applications in stem cell biology and regenerative medicine.

## Results

### High content screening for pre-selected biomimetic liver fibrotic niches

**Figure 1:**
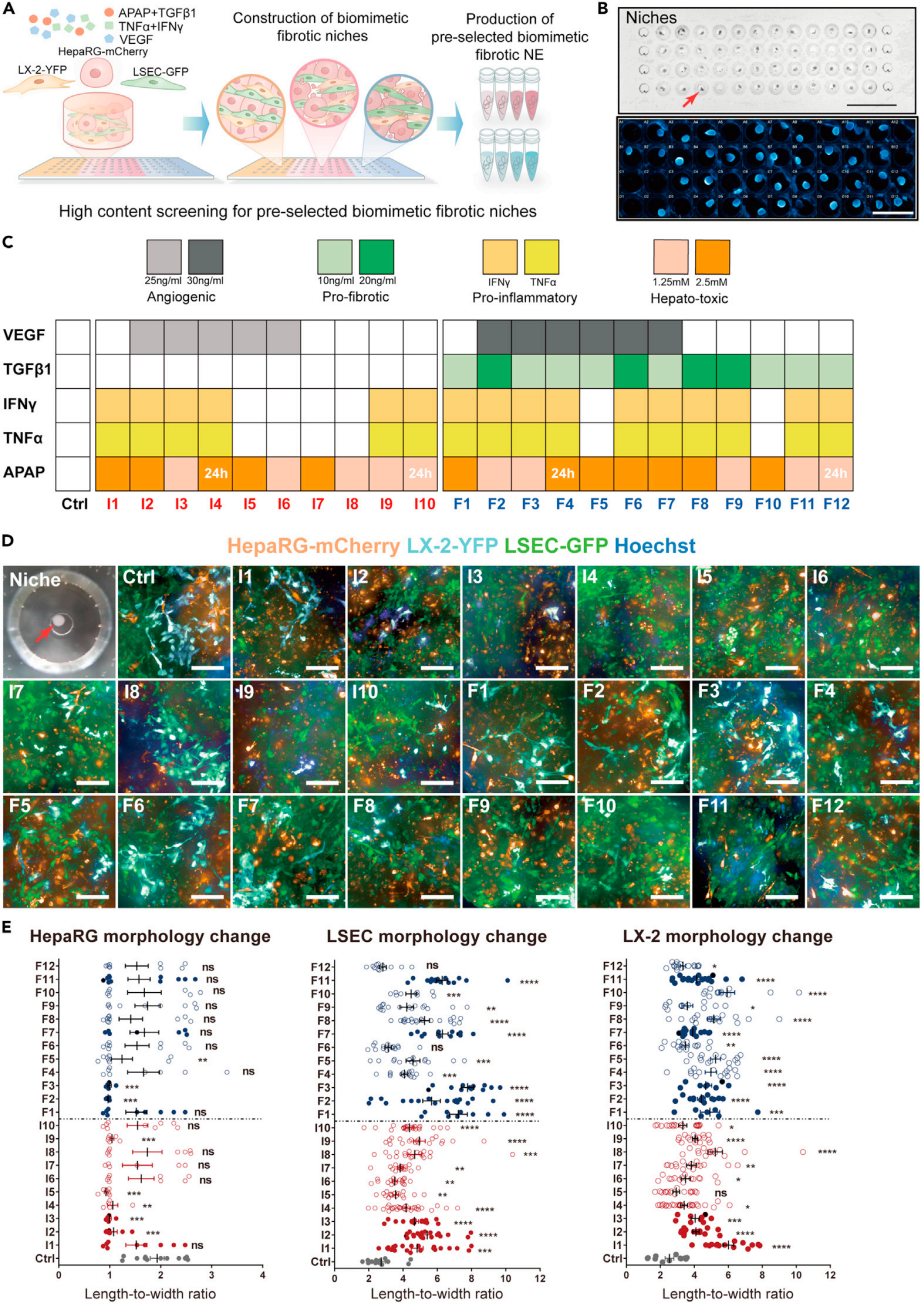
High content screening for pre-selected biomimetic liver fibrotic niches based on morphological changes (A) Schematic illustration of high content screening for biomimetic fibrotic niches and NE production. (B) (left) 3D fibrotic microtissues formed by three hepatic cell lines exposed to different combinations of pro-fibrotic factors: the red arrow indicates the location of the 3D microtissues with scale length of 10 mm; (right) high content imaging of 3D microtissues stained by Hoechst to show their shape, with scale length of 4 mm. (C) Administration of different pro-fibrotic factors to simulate the liver injury (I1-I10) and liver fibrosis (F1-F10) in the biomimetic niches. (D and E) Representative images of the biomimetic niches subjected to high content screening and the corresponding cell morphology change (length-to-width ratio). Pre-selected niches were marked with solid circles in E. Scale bar, 100 μm. Bars represent mean ± SEM. ns = not significant, ∗p < 0.05, ∗∗p < 0.01, ∗∗∗p < 0.001, ∗∗∗∗p < 0.0001.

To represent the pivotal cellular components in liver fibrotic niche, 3 types of fluorescent-labeled hepatic cell lines (i.e., hepatocyte: HepRG-mCherry, HSC: LX-2-YFP and LSEC-GFP) were chosen to visualize the morphological change inside the pathological niche. Collagen type I, as the main ECM component in the fibrotic liver, was employed as the basic matrix ingredient for the following 3D biomimetic fibrotic niche establishment based on the microwell chips previously developed by our group (Liu et al., 2017; Zhao et al., 2015) (Figures 1A and 1B). In addition, 4 types of pro-fibrotic factors were supplemented in different combinatory ratios to generate a series of biomimetic fibrotic niches with varied fibrotic status (Figure 1A). Uniform and consistent cellular contraction facilitated the niche formation (Figure 1A and Video S1). Since the hepatocyte injuries could result in liver fibrosis (Friedman, 2003), I1 to I10 represent the ‘injured’ hepatic niches induced by hepatotoxins without addition of the fibrogenic factor TGF β1, while F1 to F12 stand for the ‘fibrotic’ hepatic niche by directly including various dosages of TGF β1 (Figure 1C). Different combinations of pro-fibrotic factors that have varied modes of action were introduced into the biomimetic niches: APAP induces mitochondrial oxidative stress and acute liver injury (Leite et al., 2016); TGF-β mediates the activation of HSCs (Bataller and Brenner, 2005); inflammatory cytokines TNF-α and IFN-γ contribute to the progression of liver inflammation and fibrosis (Bataller and Brenner, 2005); and VEGF induces hepatic endothelial cell angiogenesis (Poisson et al., 2017).

Video S1. Primed 3D micro-niche formation, related to Figure 1

Hepatocytes treated by those pro-fibrotic factors became injured accompanied by an increase in cell body roundness while HSCs and LSECs were activated with a rise in length-to-width ratio to transform into myofibroblasts and undergo capillarization respectively (DeLeve, 2015; Tanaka and Miyajima, 2016). Therefore, to directly examine the different responses of the constructed 3D niche to the pathological inductions, image-based high-content screening was performed to calculate the cell body length-to-width ratio (Figures 1C and 1D). Increase in the length-to-width ratio of either LX-2 or LSECs could be an indication of success in fibrotic niche construction. Therefore, 3 conditions (I1, I2 and I3) out of the 10 ‘injured’ groups and 5 conditions (F1, F2, F3, F7 and F11) out of the 12 ‘fibrotic’ groups have been identified as ‘pre-selected fibrotic niche’ (Figure 1D). These data showed that the high content screening could serve as the initial screening for niches with fibrotic features constructed from abundant ‘input’ of pro-fibrotic factors.

### Evaluation of synthetic NE derived from pre-selected fibrotic niches based on phenotype maintenance of hESC-derived HBs

**Figure 2:**
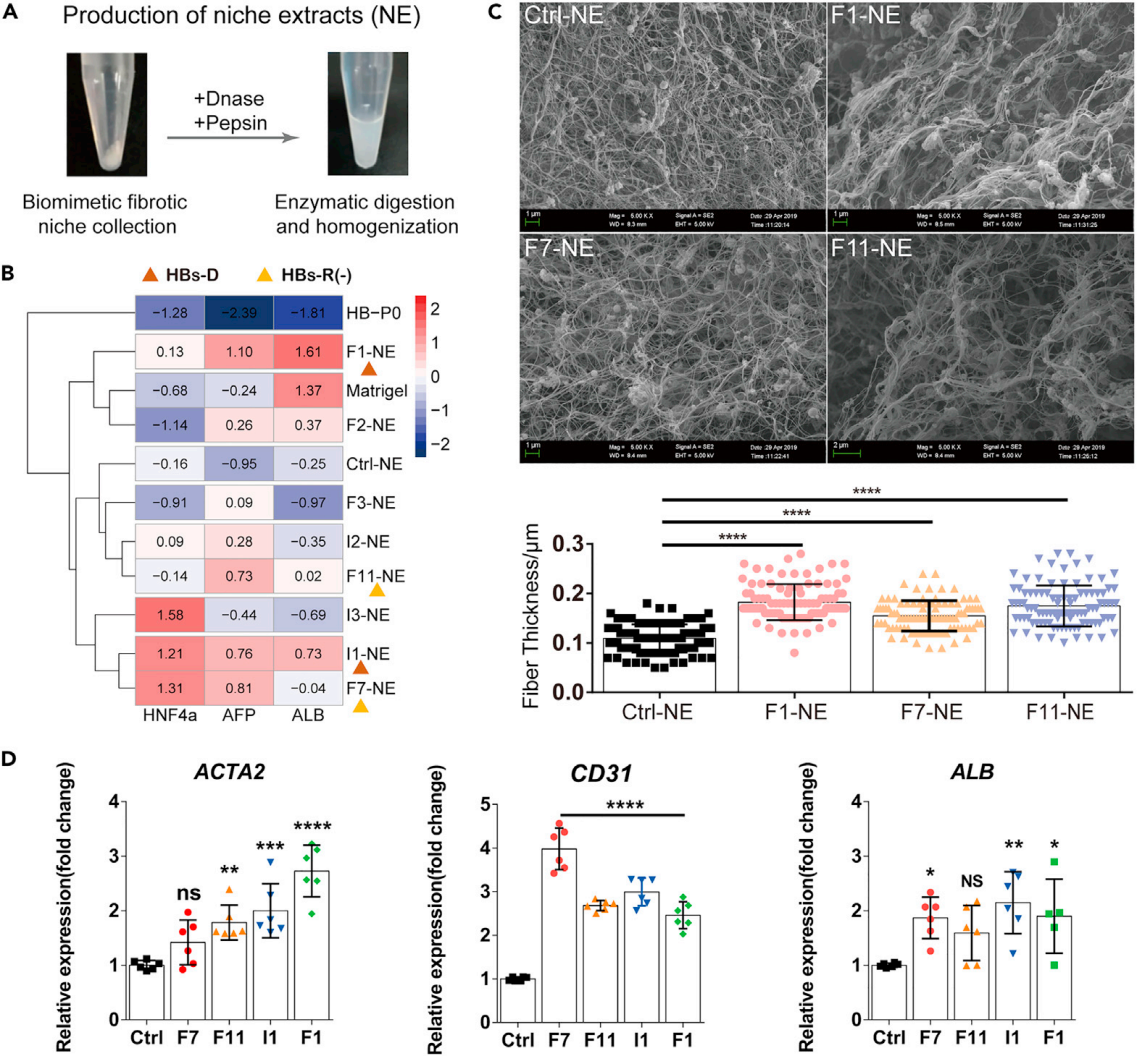
Phenotype maintenance of hESC-derived HBs on pre-selected fibrotic NEs (A) Homogeneous and stable NE production by biomimetic fibrotic niche collection, enzymatic digestion (DNase and pepsin) and homogenization until no visible debris was observed. (B) Hierarchical cluster analysis on qPCR results using *Z* score standardization to determine the expression pattern of HBs marker genes (HNF4a and AFP) and hepatic differentiation gene (ALB) in hESC-derived HBs cultured by Matrigel and different NEs. (C) SEM observation and quantification for the structure of ECM fiber bundles in different NEs. Bars represent mean ± SEM. ∗∗∗∗p < 0.0001. (D) qPCR results for the expression of *ACTA2*, *CD31* and *ALB* genes in the representative fibrotic niches, with *GAPDH* as a housekeeping gene. Bars represent mean ± SEM. ns = not significant, ∗p < 0.05, ∗∗p < 0.01, ∗∗∗p < 0.001, ∗∗∗∗p < 0.0001.

In order to produce abundant (NEs, scaling up the constructed niche from microchips to 96-well plates was realized which could maintain the morphological and gene expression features of cellular components within the niches (Figures S2A and S2B). NE production was assisted by two types of enzymatic digestions: Pepsin is applied for the production of polypeptide ingredients derived from ECM and the bioactive molecules in the niche (Tian et al., 2018); on the other hand, DNase is in charge of the removal of nucleus (Figure 2A). As primary human hepatic progenitor cells are difficult to obtain, they were substituted by hESC-derived HBs. The gene expression changes along the differentiation from hESC to HB are demonstrated in FigureS2C. To find whether the hESC-derived HBs cultured on the pre-selected NE could recapture the features of the hepatic progenitor cells, gene analysis of a panel of hepatic genes was performed (Figure 2B). EdU staining uncovered that proliferating cells were observed only in AFP+ hepatic progenitor cells, but not in cells labeled with hepatic functional marker ALB (Figure S2D). In addition, published articles demonstrate that up to 67% of newly formed hepatocytes originate from a population of HNF4a+ hepatic progenitor cells residing in the periportal region during mild fibrosis stage (Font-Burgada et al., 2015; Williams et al., 2014). Therefore,we regard hepatic progenitor cells with high expression of AFP and HNF4a as being capable of self-renewal, while the ones with high expression of ALB as being inclined toward differentiation. According to gene expression analysis, F7 NE showed superiority over its counterparts in maintaining HBs phenotype with upregulated *HNF4a* and *AFP*, and relatively low expression of *ALB*, which were labeled as ‘partially self-renewal HBs or HBs-R (−)’ (Figure 2B). In contrast, F1, I1, and F11 NE tended to promote HBs differentiation with higher expression of ALB gene, which were labeled as ‘HBs-D’ (Figure 2B). HBs naturally formed uniform colonies on Matrigel and 4 types of NE (i.e., F7 NE, F11 NE, F1 NE, and I1 NE), while organized into more heterogeneous populations on the other NE (Figure S2E).

Interestingly, all the pre-selected fibrotic NE exhibited thicker bundle of matrix fibers compared with the control NE (Figures 2C and S3), which was consistent with the increased ECM deposition and denser fiber formation in the fibrotic liver tissues. Gene expression analysis showed that compared with Ctrl niche, I1, F1, F7, and F11 niches exhibited higher expression of angiogenesis-related gene *CD31*, higher expression of myofibroblast activation-related gene *ACTA2*, and higher expression level of hepatic functional gene *ALB* (Figure 2D). The slight up-regulation of ALB expression indicates the proliferation and differentiation of HepRG in the niches, which recaptures the *in vivo* massive proliferation and subsequent differentiation of liver progenitor cells during mild fibrosis (Tanaka and Miyajima, 2016).

These data verified the success in fibrotic niche construction and the feasibility of applying NE as a liver fibrotic niche-specific reservoir for HBs *in vitro* culture.

### Optimization of the biomimetic fibrotic niches based on fibrotic gene expression and HBs phenotype maintenance

**Figure 3:**
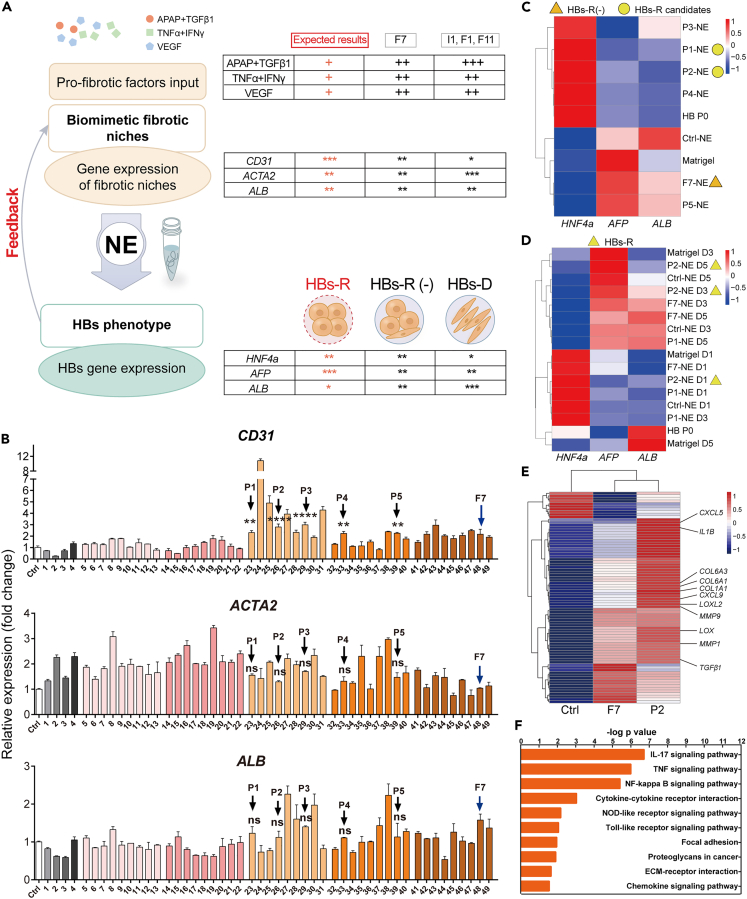
Optimization of the biomimetic fibrotic niches based on fibrotic gene expression and HBs phenotype maintenance (A) Procedures for construction of the optimal fibrotic niches: Correlative analysis of the profibrotic factors input, the gene expression of the fibrotic niches as well as the HBs phenotypes (gene expression) retained on corresponding NEs could guide prediction of the input factors and the fibrotic niche features resulting in improved HBs phenotype maintenance (HBs-R). (B) qPCR results show the expression of *ACTA2*, *CD31*, and *ALB* in fibrotic niches constructed from 50 combinations of profibrotic factors; blue arrows refer to the F7 niche (representative HBs-R (−) niche), which serves as a reference for optimization. Five HBs-R NE candidates (i.e., P1-P5) were marked; Concentrations of the compounds and factors shown on the horizontal axis are detailed in the Table S1, with *GAPDH* as housekeeping gene. Bars represent mean ± SEM. ns = not significant, ∗∗p < 0.01, ∗∗∗p < 0.001, ∗∗∗∗p < 0.0001. (C) Hierarchical cluster analysis of qPCR results for *HNF4a*, *AFP* and *ALB* gene expression of HBs cultured on the five HBs-R NE candidates for 3 days. (D) Hierarchical cluster analysis of qPCR results for gene expression of HBs cultured on the P1 NE, P2 NE, F7 NE and Matrigel for day 1, day 3, and day 5 to select the P2 NE as the representative HBs-R NE. (E) RNA sequencing analyzed the difference in gene expression between P2 niche and F7 niche. Differentially expressed (p < 0.01 and fold change>2) and top 50 ranked genes from P2/Ctrl and F7/Ctrl respectively were examined by hierarchical cluster analysis, and the difference in differentially highly expressed genes from P2/Ctrl versus F7/Ctrl was demonstrated as a heatmap. The fibrosis-related genes were labeled in the heatmap. (F) KEGG signaling pathway enrichment analysis on RNA-seq results for 229 significantly highly expressed genes in synthetic P2 niche when compared with both the control niche and F7 niche with the IL-17 signaling as the most enriched pathway.

To further optimize the fibrotic NE that could outperform the F7 NE in maintenance of HBs self-renewal phenotype (HBs-R), optimization was performed through correlative analysis of the pro-fibrotic factors input, the gene expression of the fibrotic niches, and the HBs phenotypes (gene expression) retained on corresponding NE (Figure 3A). Gene expression analysis showed that compared with F1, I1, and F11 niches (whose NE induce HBs-D), F7 niche [whose NE induce HBs-(R-)] exhibited higher expression of angiogenesis-related gene *CD31*, lower expression of myofibroblast activation-related gene *ACTA2*, and a higher expression level of hepatic functional gene *ALB* (Figure 2D). Based on the feedback from the correlative analysis, we predicted that a lower dose of profibrotic factors could result in optimized fibrotic niches with relatively a lower level of *ACTA2* and a higher level of *CD31* gene expression compared with the F7 niche whose NEs were expected to realize better phenotype maintenance and expansion of HBs *in vitro* (HBs-R) (Figure 3A). Fifty fibrotic niches were constructed based on 50 combinations of profibrotic factor input with reduced concentrations compared to the factor input used to construct the F7 niche (Table S1). According to the above-mentioned criteria in niche gene expression, five HBs-R niche candidates were screened out (listed as P1-P5 niche) which exhibited higher expression levels of *CD31*, and similar expression levels of *ACTA2* and *ALB* as compared with the F7 niche (Figures 3B and S4A). Likely, phenotype maintenance of the hESC-derived HBs cultured on P1-P5 NE as well as the ‘Matrigel’ were examined by hierarchical clustering of the gene expression of *HNF4a*, *AFP*, and *ALB* (Figure 3C). 2 out of the 5 NEs (i.e., P1 NE and P2 NE) exhibited pro-self-renewal capabilities for HBs. In particular, the P2 NE outperformed F7 NE and Matrigel for HBs phenotype maintenance for 5 days (Figure 3D).

Bulk RNA-seq analysis showed the up-regulated expression of key fibrotic genes (e.g., *TGFB1*, *MMP1*,*9*; *LOX*, *LOXL2*, *COLL1A1*) in the P2 niche compared with the F7 niche, which could be categorized into growth factors, growth factor receptors, ECM, matrix proteases, angiogenesis, and interferon signaling pathways (Figures 3E and S4B). Remarkably, the top up-regulated KEGG pathways fell on the Interleukin-17 signaling pathway (Figure 3F), which includes members that take part in the liver fibrogenesis such as IL-17A. IL-17 is a contributor to tissue injury through production of reactive oxygen species (ROS) by potentiating neutrophil responses and a facilitator of ECM production through increasing the expression of TGF-β receptors on fibroblasts (Henderson et al., 2020). Other top-enriched KEGG pathways included TNF, NF-κB, PI3k/Akt, NOD and Toll-like receptor signaling pathways (Figure 3F), which all play critical roles in liver fibrosis (Aoyama et al., 2010; Wang et al., 2015; Yang and Seki, 2015).

Those results implied that the P2 niche, induced by a lower dose of profibrotic factors, was proved to better simulate the *in vivo* fibrotic microenvironment and the corresponding P2 NE could be applied as optimal matrix substrate for HBs phenotype maintenance.

### Synthetic fibrotic NE promoted phenotype maintenance of hESC-derived HBs and angiogenesis of hESC-derived ECs in 2D culture

**Figure 4:**
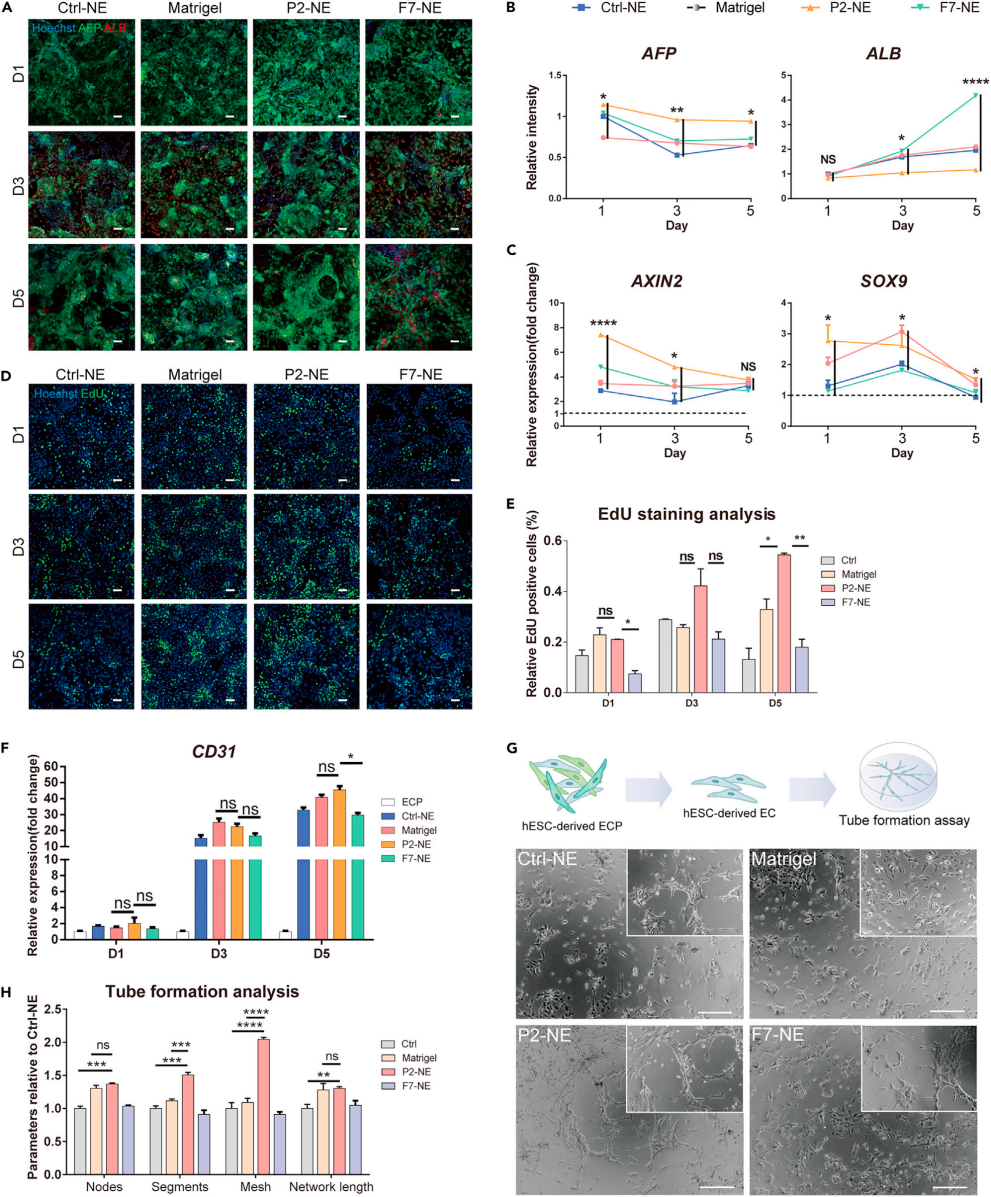
Synthetic fibrotic NE promoted phenotype maintenance of hESC-derived HBs and angiogenesis of hESC-derived ECs in 2D culture (A and D) AFP/ALB immunofluorescence and EdU staining of hESC-derived HBs cultured on plate coated with Matrigel and different NE on day 1, day 3, and day 5, with a scale bar of 100 μm. (B) Quantitative statistics of AFP/ALB immunofluorescence staining. Bars represent mean ± SEM. ns = not significant, ∗p < 0.05, ∗∗p < 0.01, ∗∗∗∗p < 0.0001. (C) Gene expression of self-renewal genes *AXIN2* and *SOX9* of hESC-derived HBs cultured on Matrigel and different NEs on day 1, day 3, and day 5, with GAPDH as housekeeping gene. Bars represent mean ± SEM. ns = not significant, ∗p < 0.05, ∗∗∗∗p < 0.0001. (E) Quantitative statistics of EdU staining for cell proliferation. Bars represent mean ± SEM. ns = not significant, ∗p < 0.05, ∗∗p < 0.01. (F) qPCR results show the expression of endothelial marker gene *CD31* of hESC-derived endothelial progenitor cells cultured on Matrigel and different NEs at day 1, day 3, and day 5. Bars represent mean ± SEM. ns = not significant, ∗p < 0.05. (G) Schematic representation and phase contrast images of hESC-derived endothelial progenitor cells cultured on Matrigel and different NEs. Scale bar is 200 μm. (H) Tube formation analysis of endothelial cells on Matrigel and different NEs. Bars represent mean ± SEM. ns = not significant, ∗∗p < 0.01, ∗∗∗p < 0.001, ∗∗∗∗p < 0.0001.

To further investigate whether the synthetic fibrotic P2 NE would recapitulate featured cellular responses during fibrosis, hESC-derived HBs and endothelial cell progenitors (ECPs) were cultured on the tissue culture plates coated with NE as compared with Matrigel coating. Sustained *AFP* expression (Figures 4A and 4B), elevated expression of key self-renewal markers (i.e., *AXIN2* and *SOX9)* (Figure 4C) and enhanced proliferation (Figures 4D and 4E) of HBs during 5 days *in vitro* culture implied that the P2 NE could promote self-renewal of HBs. As reported, angiogenesis is a pathological manifestation of liver fibrosis and capillarization of liver sinusoid endothelial cells (LSECs) that has been observed in parallel with fibrogenesis in chronic liver disease (Lee et al., 2007; Zhang et al., 2017). The P2 NE-coated 2D substrate was shown to promote endothelial differentiation of hESC-derived ECPs into mature endothelial cells (ECs) during 5 days’ culture with significant increase in the expression of endothelial marker *CD31* (Figure 4F). The intensified angiogenesis of ECPs was further verified via the tube formation assay where more established vascular structures were observed in the P2 NE group, compared with the F7 NE and Matrigel groups (Figures 4G and 4H). Taken together, these results demonstrate that P2 is superior to F7 and marginally better than Matrigel in terms of promoting phenotype maintenance in hESC-derived HBs and angiogenesis of hESC-derived ECs in 2D culture.

### Synthetic fibrotic NE facilitated 3D liver organoids formation with recaptured fibrotic features

**Figure 5:**
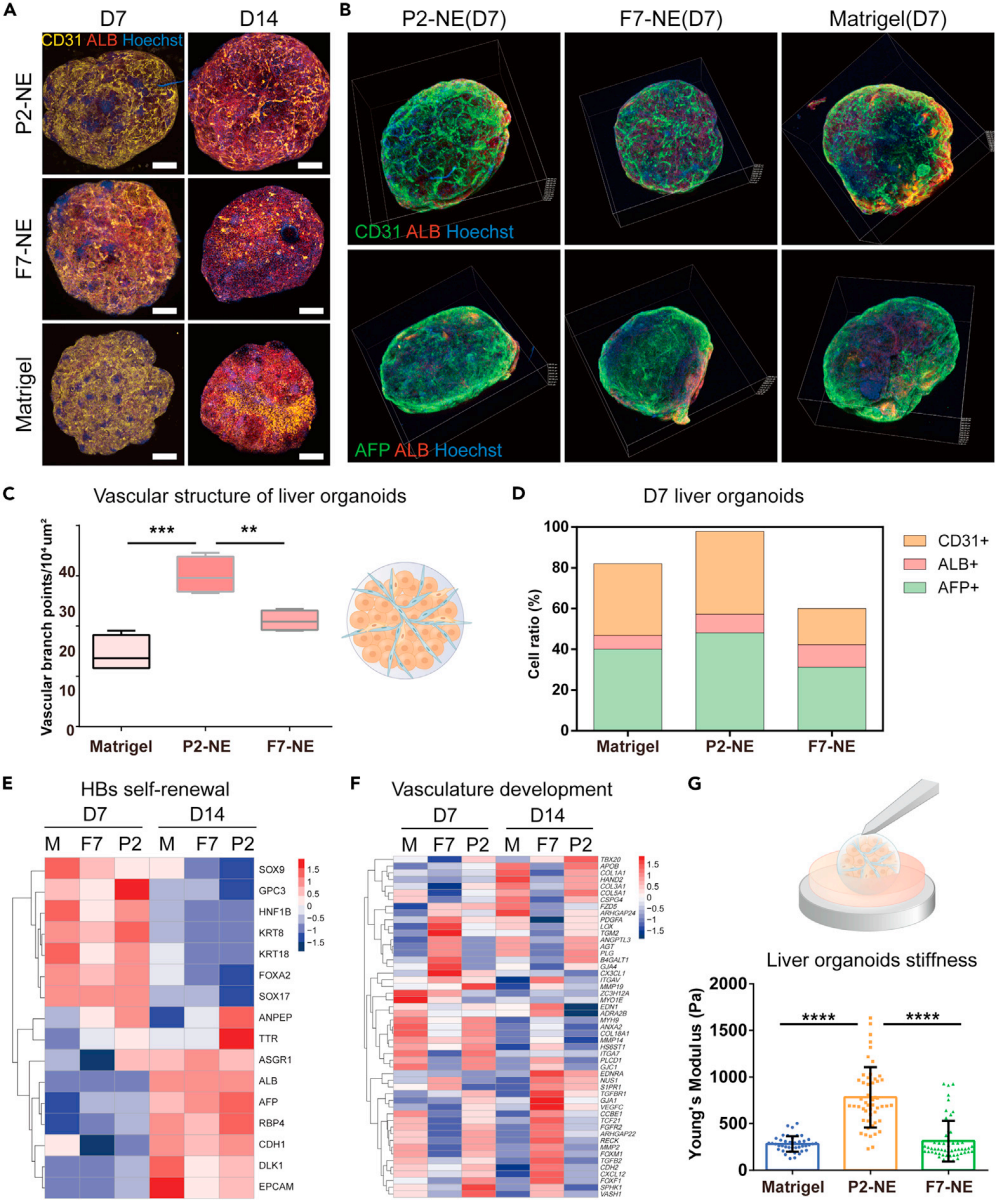
Synthetic fibrotic NE enhanced liver fibrotic features in 3D liver organoids (A) Stacked confocal immunofluorescence images of 3D liver organoids derived from hESC-derived HBs, hESC-derived endothelial progenitor cells, and UC-MSC co-cultured within Matrigel, P2 NE, and F7 NE with vascular structure stained by CD31 on day 7 and day 14 respectively, Scale bar: 200 μm. (B) Tissue clearing-enhanced 3D visualization of the immunofluorescence staining for CD31 (vascular structure), AFP (HBs self-renewal), and ALB (HBs differentiation) of Matrigel, P2 NE, and F7 NE-assisted 3D liver organoids after 7 days’ culture. (C) Quantitative analysis for the number of vascular branch points in the vascular structure in (B) determined by ImageJ. Bars represent mean ± SEM. ∗∗p < 0.01, ∗∗∗p < 0.001. (D) Cell ratio analysis of the AFP+, ALB+ and CD31+ cells within the 3D liver organoids after 7 days’ culture. (E and F) Cluster analysis of RNA-seq genes related to HBs self-renewal and vascular development of 3D organoids derived from Matrigel, P2 NE and F7 NE after 7 days’ and 14 days’ culture. (G) Determination of 3D organoid stiffness after 7 days’ culture by AFM. Bars represent mean ± SEM. ∗∗∗∗p < 0.0001.

Since 3D culture configuration out-performed its 2D counterpart with closer resemblance to the natural niche, 3D liver organoids were constructed with 3 precursor cell types, namely hESC-derived HB, hESC-derived ECP, and inactivated human umbilical cord derived mesenchymal stem cells (UC-MSCs), which were co-cultured within NE or Matrigel. Extensive vascularization was observed in the P2 NE-derived 3D organoids in 7 days which could be stabilized up to 14 days. In contrast, the vascular structures were gradually diminished from 7 days to 14 days in the F7 NE and Matrigel-derived 3D organoids (Figure 5A). To facilitate 3D visualization and quantitative analysis of the immune-stained organoids, tissue clearing was performed (Video S2. Tissue clearing-enhanced visualization of Matrigel based organoids immune-stained by CD31, related to Figure 5, Video S3. Tissue clearing-enhanced visualization of F7 NE based organoids immune-stained by CD31, related to Figure 5, Video S4. Tissue clearing-enhanced visualization of P2 NE based organoids immune-stained by CD31, related to Figure 5), which confirmed the enhanced vascular formation in the P2 NE-derived 3D organoid (Figures 5B and 5C). Meanwhile, elevated expression of AFP and CD31 was achieved in the P2 NE-derived 3D organoid compared to the F7 NE and the Matrigel counterparts as revealed by immunostaining and cell ratio assay (Figures 5B and 5D).

Video S2. Tissue clearing-enhanced visualization of Matrigel based organoids immune-stained by CD31, related to Figure 5

Video S3. Tissue clearing-enhanced visualization of F7 NE based organoids immune-stained by CD31, related to Figure 5

Video S4. Tissue clearing-enhanced visualization of P2 NE based organoids immune-stained by CD31, related to Figure 5

Next, bulk RNA-Seq analysis of the organoids revealed that the sixteen genes related to HBs self-renewal (e.g., *AFP*, *TTR*, *RBP4*, *DLK1*, *GPC3*, *FOXA2*, *SOX17*, and *KRT8*) were relatively at a higher expression level in the P2 NE-derived organoid at day 7 and day 14 compared with the F7 NE and Matrigel counterparts (Figure 5E). Similarly, the expression of the 51 genes related to the vascular development was relatively higher in P2 NE-derived organoids (Figure 5F). Additional q-PCR analysis was conducted to confirm the up-regulated gene expression of AFP and CD31 and down-regulated expression of hepatic differentiated genes (i.e., ALB, RBP4) (Figure S5). Up-regulation of E-Cadherin in the P2 NE-derived 3D organoids was also observed indicating enhanced cell-cell interactions (Figure S5). Interestingly, the biomechanical properties of the 3D organoids could be modulated with increased Young’s modulus (∼800Pa) of the P2 NE-derived 3D organoids compared with the F7 NE and Matrigel counterparts (∼300Pa), recapitulating the rise in tissue stiffness during liver fibrosis *in vivo* (Figure 5G).

In summary, HBs self-renewal and vascular structural features in liver fibrogenesis was recreated in the P2 synthetic NE-derived 3D organoids suggesting the biomimicry of P2 fibrotic niche and the corresponding P2 NE.

### Defined ECM and factors combination resolved from the P2 NE promoted *in vitro* HBs expansion

**Figure 6:**
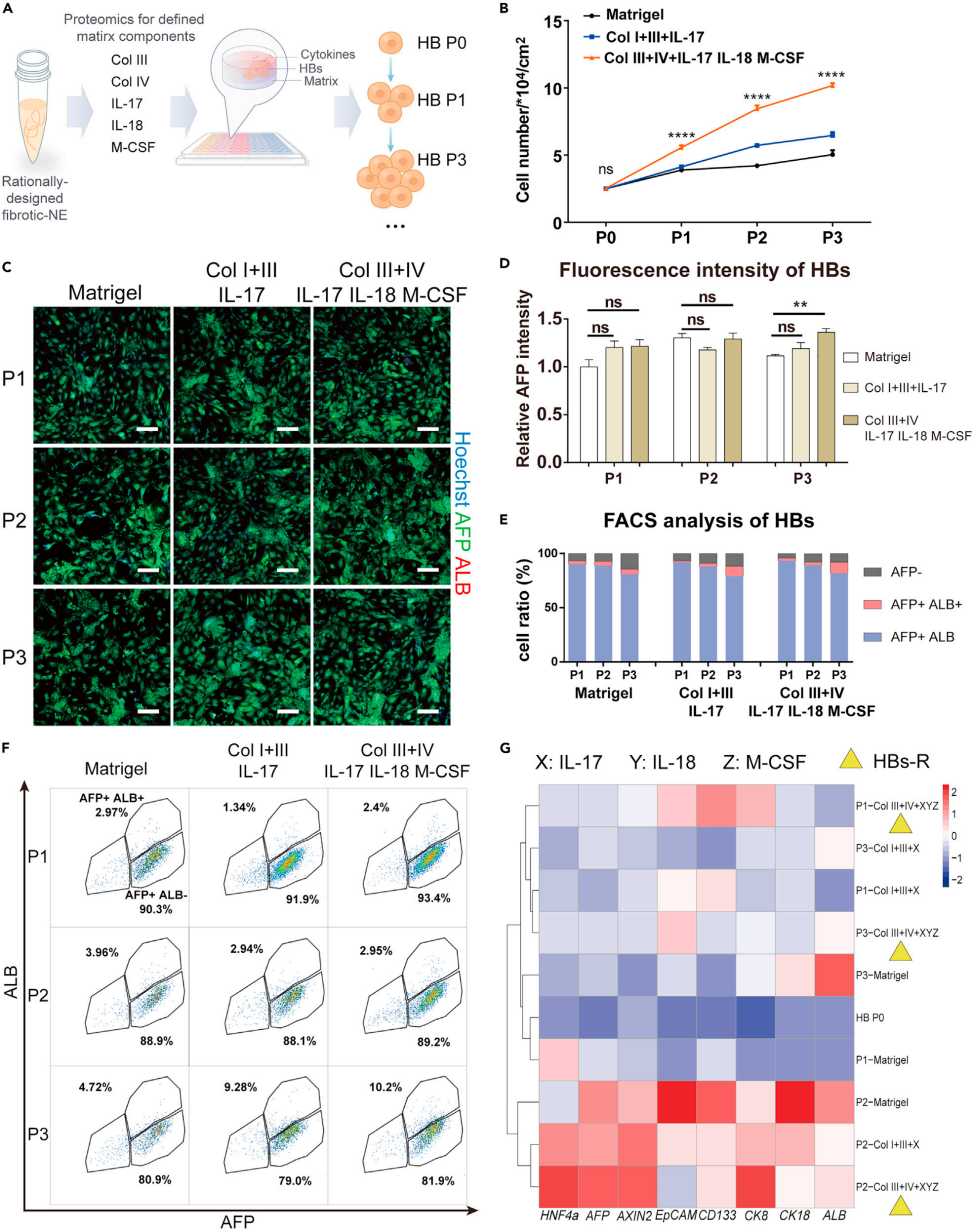
Defined ECM and factors combination resolved from fibrotic NE enhanced hESC-derived HBs expansion *in vitro* (A) Schematic representation of resolving ECM and factor combinations from NE to enhance multi-passaged HBs phenotype maintenance and expansion *in vitro*. (B) Cell count for hESC-derived HBs cultured on the 3 different substrates (i.e., Matrigel, Col I + III + IL-17 and Col III + IV + IL-17+IL-18+M-CSF) for three passages. Bars represent mean ± SEM. ∗∗∗∗p < 0.0001. (C and D) Immunofluorescence staining and the corresponding intensity analysis of AFP and ALB in hESC-derived HBs cultured on different substrates for three passages, scale bar: 100 μm. Bars represent mean ± SEM. ns = not significant, ∗∗p < 0.01. (E and F) Flow cytometry analysis and the related quantification of the ratio of AFP^high^/ALB^low^ (AFP + ALB-, self-renewal HBs) and AFP^high^/ALB^high^ (AFP + ALB+, differentiated HBs) cell population after 3 passages of hESC-derived HBs (HB P0) on the three substrates; (G) Cluster analysis for results of qPCR on the expression of HBs marker genes (*HNF4a*, *AFP*, *AXIN2*, *EpCAM*, *CD133*, *CK8* and *CK18*) and HBs differentiation gene (*ALB*) on different substrates, gene expression difference is demonstrated as heatmap.

HBs have been proposed as promising candidates for cell-based therapy of chronic liver diseases (Chen et al., 2020; Fu et al., 2019). To facilitate the understanding of NE and their application for HBs expansion, we attempted to resolve the ECM components and factors in the P2 NE which contributed to the HBs phenotype maintenance and expansion, in order to reconstitute the fibrotic niches with defined NE components (Figure 6A).Quantitative proteomics analysis for the P2 and F7 NE ingredients indicated significant up-regulation of 2 types of ECM components (i.e., collagen type III and collagen type IV) and 2 types of inflammatory factors (i.e., IL-18 and M-CSF) (Table S2). As previously shown by RNA-seq analysis (Figures 3E and S6A), IL-17 signaling pathway was up-regulated in the fibrotic niche and IL-17 treatment was reported to promote proliferation of bipotential murine oval cells—a liver progenitor-like cell (Guillot et al., 2018). Therefore, IL-17 was also introduced into the combinatorial groups of the defined ECM and factors for HBs expansion. Immunostaining and hierarchical clustering based on the expression of self-renewal markers suggested the effectiveness of three combinations toward HBs phenotype maintenance and expansion: collagen type III+ IL-17, collagen type III+ type IV + IL-18+M-CSF, and collagen type III+ type IV + IL-17+IL-18+M-CSF (Figures S6B–S6D). Collagen type I was also included as the major ECM component in the liver and the basal ECM for construction of *in vitro* fibrotic niches. Immunofluorescence staining and gene expression analysis both showed the superiority of Col III + IV + IL-17+IL-18+M-CSF to Col I + III + IL-17 for HBs phenotype maintenance with higher expression of *HNF4A*, *AFP*, and *AXIN2* and relatively lower expression of *ALB* (Figure S7).

Next, we investigated the influence of the defined niche components for multi-passage of HBs. Col III + IV + IL-17+IL-18+M-CSF based substrate could achieve the HBs expansion from an initial cell number of 2.5∗10^4^ to 10.2∗10^4^ after three passages, which outperformed the Matrigel and Col I + III + IL-17 based substrates (Figure 6B). Meanwhile, the Col III + IV + IL-17+IL-18+M-CSF expanded HBs exhibited higher AFP expression by immunostaining (Figures 6C and 6D), which was comprised of a ratio of AFP + ALB- cell population comparable to that of Matrigel as revealed by flow cytometry (Figures 6E and 6F). q-PCR analysis for HBs upon 7 passages expansion was conducted to confirm the advantage of Col III + IV + IL-17+IL-18+M-CSF in promoting up-regulation of hepatoblasts gene markers (i.e., *HNF4a*, *AFP*, *EpCAM*, *CD133*, *CK8*, and *CK18*) (7) and self-renewal marker (i.e., *AXIN2)* as well as down-regulation of *ALB* (Figure6G), although this advantage gradually diminishes with repetitive passages (Figure S8).

Finally, hESC-derived HBs expanded on Matrigel or collagen III+ type IV + IL-17+IL-18+M-CSF for 3 passages exhibited similar hepatic differentiation potency as verified by immunostaining of hepatic functional markers of CYP3A4 and ALB (Figure S9A), expression of hepatocyte genes (i.e., *CYP3A4*, *CYP2C9*, *CYP2C19*, *αAT* and *ALB*) and urea production (Figures S9B and S9C). Taken together, the defined ECM and factors combination resolved from fibrotic niche could replace the P2 NE for multi-passaged *in vitro* expansion of hESC-derived HBs, which exhibited potency to undergo hepatic differentiation *in vitro*.

## Discussion

This study screened biomimetic liver fibrotic niches and developed a novel method of acquiring *in vitro* fibrotic bioactive ingredients, namely NE. The NE could retain most of the intracellular ingredients except the nucleus and extracellular ECM/factors. The synthetic NE could achieve HBs phenotype maintenance and promote vascularization of ECs under both 2D culture and 3D organoid configurations, which recapitulate the hepatic progenitor cell expansion and angiogenesis in the early stages of liver fibrosis *in vivo*. Additionally, our study highlighted the advantage of the defined components (i.e., collagen type III, collagen type IV, IL-17, IL-18 and M-CSF) resolved from the NE over Matrigel for *in vitro* HBs expansion.

To the best of our knowledge, it is the first time that bioactive ingredients derived from *in vitro* constructed biomimetic pathological niches are applied for hepatic progenitor cell expansion and 3D liver organoid formation. It should be noted that the screened NE is only marginally better than Matrigel at phenotype maintenance of hESC-derived HBs and recapitulation of the pathological angiogenesis of hESC-derived ECs both in 2D culture and 3D liver organoids. However, Matrigel is not suitable to assist cell and organoid transplantation in clinical therapy due to its tumorigenic origin and batch-to-batch variability. In contrast, by further identifying key proteins and factors in the NE, the current study can potentially offer defined and medical-grade ECMs for applications toward *in vivo* cell or organoid transplantation in clinical setting. Furthermore, by implementing our proposed method, individualized and customized NE is rendered possible, which broadens their applicability.

Although there have been numerous 3D liver fibrosis *in vitro* models, most of them were far from being able to faithfully recapitulate the complexity and pathological features of the *in vivo* counterparts. Here, we applied three key hepatic cell types to establish the 3D liver fibrosis model, with the addition of inflammatory factors that potentially act as effective substitution to Kupffer cells participating during the fibrosis progression. Nevertheless, Kupffer cells are capable of secreting multiple inflammatory factors (e.g., TNFα, IL-6, and IL-1β) that trigger fibrosis, followed by recruitment of monocyte-derived macrophages that tuned the fibrogenesis. The role of Kupffer cells and monocyte-derived macrophages as well as their waxing and waning in the fibrosis progression may not be effectively substituted by a limited number of inflammatory factors. Therefore, the introduction of Kupffer cells and monocyte-derived macrophages during the construction of *in vitro* biomimetic fibrotic niches should be accomplished in the future to improve the current biomimetic fibrotic model.

In this study, a specific gene expression pattern of hESC-derived hepatoblasts with high expression of hepatoblast markers genes (e.g., *HNF4a* and *AFP*) and relatively low expression of the differentiation gene (e.g., *ALB*) was defined as the phenotypic maintenance and expansion of hepatoblasts. This pattern can reflect the ability of HBs to both maintain their phenotype and inhibit spontaneous differentiation due to microenvironmental perturbations as well as indicating their proliferative capacity through high expression of stemness genes. We proved that the defined components from the synthetic NE, namely collagen type III, type IV, IL-17, IL-18 and M-CSF combination, could enhance the phenotype maintenance and expansion of HBs. Collagen types III and IV are both involved in the proliferation of HBs during fetal liver development and liver fibrosis (Baptista et al., 2014; McClelland et al., 2008). IL-17 signaling was proved to facilitate production of IL-6, IL-1, and tumor necrosis factor alpha (TNF-α) by immune cells and increased the expression of transforming growth factor-1 (TGF-1), which is a fibrogenic cytokine (Henderson et al., 2020). In addition, IL-17 directly induced production of collagen type I in HSCs by activating the signal transducer and activator of transcription 3 (Stat3) signaling pathway (Meng et al., 2012). Both IL-18 and M-CSF are involved in liver pathology. IL-18, a cytokine in the IL-1 family, is produced in the liver by Kupffer cells stimulated by microbial antigens such as LPS, which contributes to fibrogenesis by inducing macrophages to overexpress inflammatory cytokines (Dinarello, 1999). M-CSF can be secreted by many cell types, such as monocytes/macrophages, fibroblasts, bone marrow stromal cells, lymphocytes, and endothelial cells. In acute or chronic liver fibrosis and cirrhosis, elevated serum levels of M-CSF secreted by infiltrating mononuclear macrophages may represent ongoing hepatocellular necrosis (Itoh et al., 1994). Future studies are needed to dissect the regulatory mechanisms of these matrices and cytokines on HBs fate determination.

Since the stem cell microenvironment is a highly sophisticated community comprising of multiple cell types and complex biochemical/biophysical factors, the synergistic effects of biophysical factors (e.g., hypoxia and biomechanics) should be considered in the niche construction and their influences on stem cell fate need to be further explored. In addition to the biochemical components, we also preliminarily investigated the effects of hypoxia and substrate stiffness on HBs culture. It was found that hypoxia with 5% oxygen (Figure S10) and an appropriate substrate Young’s modulus (3.5 kPa) simulating the tissue mechanics of early-stage hepatic fibrosis (Guo et al., 2020) were conducive to the maintenance of phenotype and expansion in HBs, which shed light on the involvement of multifaceted biochemical and biophysical cues in future exploration for niche optimization.

### Limitations of the study

The screened NE is only marginally better than Matrigel at phenotype maintenance of hESC-derived HBs and recapitulation of the pathological angiogenesis of hESC-derived ECs both in 2D culture and 3D liver organoids. More efforts are needed to screen out NE that is more suitable for expansion of HBs. We applied the 3 critical hepatic cell types, except for macrophages, to improve the 3D liver fibrosis model, with the addition of inflammatory factors that potentially act as effective substitution for Kupffer cells participating during the fibrosis progression. However, Kupffer cells are capable of secreting multiple inflammatory factors (TNFα, IL-6, IFNγ and IL-1β) that trigger fibrosis, followed by recruitment of monocyte-derived macrophages that contribute to the ongoing development of fibrosis; the role of Kupffer cells and monocyte-derived macrophages and their waxing and waning dynamic changes in the fibrosis development may not be effectively substituted by the addition of only a limited number of inflammatory factors. Therefore, the introduction of Kupffer cells and monocyte-derived macrophages to participate in different segments of the fibrosis process during the construction of *in vitro* biomimetic fibrotic microenvironment is a future research direction.

## STAR★Methods

### Key resources table

**Table:** 

REAGENT or RESOURCE	SOURCE	IDENTIFIER
**Antibodies**		

Mouse monoclonal anti-AFP	R&D	Cat# MAB1368; RRID:AB_357658
Rabbit monoclonal anti -ALB	Abcam	Cat# ab207327; RRID:AB_2755031
Mouse monoclonal anti--CD31	Abcam	Cat# ab9498; RRID:AB_570940

**Chemicals, peptides, and recombinant proteins**		

human Activin A	Peprotech	Cat#120-14E
human BMP4	Peprotech	Cat#120-05
human bFGF	Peprotech	Cat#100-18B
human OSM	Peprotech	Cat#300-10
human EGF	Peprotech	Cat#100-55B
human HGF	Peprotech	Cat#100-39H
human VEGF	Peprotech	Cat#100-20-250
human IL17A	Peprotech	Cat#200-17
human IL18	Novoprotein	Cat#Q14116
human MCSF	Peprotech	Cat#300-25
human collagen III	Sigma	Cat#CC054
human collagen IV	Sigma	Cat#AB748
mouse collagen I	BD	Cat#354236
CHIR99021	Sigma	Cat#SML1046
Dex	Sigma	Cat#D4902
L-Ascorbic acid	Sigma	Cat#A8960
Y27632	MedChemExpress	Cat#HY-10583
Insulin	Biological Industries	Cat#41-975-100
Nicotinamide	Sigma	Cat#N0636

**Critical commercial assays**		

CellTiter-Blue®Cell Viability Assay	Promega	Cat#G8080
Enhanced BCA Protein Assay Kit	Beyotime	Cat#P0010
TRIzol RNA isolation reagents	Invitrogen	Cat#15596018
Visikol HISTO-M Starter kit	Sigma	Cat#HMSK-1
BeyoClick™ EdU Cell Proliferation Kit	Beyotime	Cat#C0071S
Urea Assay Kit	BioAssay Systems	Cat#DIUR-100

**Experimental models: Cell lines**		

human LSEC cell line	ScienCell	N/A
human LX-2 cell line	Xiangya Hospital of Centre-South University	N/A
HepaRG cell line	Xiangya Hospital of Centre-South University	N/A
h1 ESC	WiCell Institute	N/A
h9 ESC	WiCell Institute	N/A
Oligonucleotides		
Primers for quantitative RT-PCR, see Table S3	This paper	N/A

**Software and algorithms**		

CFX Manger Software	Bio-Rad	https://www.bio-rad.com/en-kr/sku/1845000-cfx-manager-software?ID=1845000
ImageJ	Version 2.1.0	https://imagej.nih.gov/ij/
Nano Measurer	Version 1.2.0.5	https://nano-measurer.software.informer.com/
FlowJo_V10	BD	https://www.flowjo.com/
GraphPad Prism	Version 7.0	https://www.graphpad.com/scientific-software/prism/
Proteome Discoverer Software	Thermo-Fisher Scientific	Cat#OPTON-30812
Python	Version 3.9.7	https://www.python.org/
RStudio	Version 1.3.1093	https://www.rstudio.com/products/rstudio/

### Resource availability

#### Lead contact

Further information and requests for resources and reagents should be directed to and will be fulfilled by the lead contact, Yanan Du (email: duyanan@tsinghua.edu.cn).

#### Materials availability

3D microchip and stable reporter cell lines generated in this study are available from the lead contact without restriction.

### Experimental model and subject details

#### Cell culture

Human LSEC cell line and human hepatic stellate cell line (LX-2) were cultured in medium composed of high glucose Dulbecco’s modified Eagle medium (4.5 g/L glucose, Wisent, Canada) supplemented with 10% FBS (Wisent) and 1% penicillin-streptomycin (Wisent). HepaRG cells were cultured in Williams’ E medium (Gibco) supplemented with 12% FBS, 1x Glutamax (Invitrogen), 0.1 μM dexamethasone (Dex) (Sigma-Aldrich), 5μg/mL insulin (Aladdin) and 1% penicillin-streptomycin.

These hESC lines (H1 and H9) were maintained in TeSR-E8 essential medium (Stemcell Technologies) in a 37°C incubator at 5% CO2 and 100% humidity and the medium was changed daily. hESC lines and hESC-derived cells were seeded onto Matrigel-coated plates (Corning). For initiation of cell differentiation, hESCs were seeded at a density of 1.2 × 10ˆ5 cells per well of a 12-well plate in E8 essential medium containing 10 μM Y27632 (MedChemExpress).

By optimization of hepatic differentiation protocol, we generated hESC-derived hepatoblasts as described below: hESCs were induced into definitive endoderm cells with the combination of 100 ng/ml Activin A (Peprotech), 3 μM CHIR99021 (MedChemExpress) and 1×GlutaMax for 3 days in RPMI1640 medium (Wisent) with B27 supplement (Invitrogen). The hESC-derived endoderm cells were further specified into hepatoblasts with 10 ng/ml BMP4 (Peprotech), 10 ng/ml bFGF (Peprotech) and 1×GlutaMax for 4 days in RPMI1640 medium with 1%KSR (Gibco). At this stage, the hepatoblasts could be expanded in the optimized hepatoblasts expansion medium: DMEM/F12 medium (Invitrogen) with 10% FBS, 20 ng/ml EGF (Peprotech), 40 ng/ml HGF (Peprotech), 10 mM Nicotinamide (Sigma-Aldrich), 1x ITS supplement (Sigma-Aldrich), 0.1 μM Dex and 1% penicillin-streptomycin.

hESC-derived endothelial progenitor cells and endothelial cells induction was optimized based on the method described by Zhang et al. (Zhang et al., 2017). The basal AATS medium patented from Prof. Na Jie’s Laboratory at Tsinghua University was replaced daily. For endothelial progenitor cells differentiation, hESCs cultured medium was changed to AATS medium with 5 ng/ml BMP4 for 1 day and AATS medium with 5 ng/ml BMP4 and 10 μM CHIR for 2 days. For endothelial cells differentiation, endodermal progenitor cells were digested into single cells with Accutase and seeded onto Matrigel-coated plate wells at a density of 2.5× 10ˆ5 cells per well for a 12-well plate in AATS medium with 50 ng/ml VEGF (Peprotech) plus 10 ng/ml bFGF (Peprotech) for 3 days.

#### Generation of stable HepaRG-mCherry, LX-2-YFP and LSEC-GFP reporter cells

Stable cell lines of HepaRG-mCherry, LX-2-YFP and LSEC-GFP were obtained through lentivirus-mediated infection and screening. Prior to infection, single cells were seeded onto Poly-lysine-coated well plates and grown to 60% confluence, following which concentrated mCherry, YFP or GFP lentivirus were added to the medium at a final concentration of 8 μg/mL polybrene supplement. After 12 hours of infection, cells were washed twice with PBS and cultured in corresponding medium. After culturing for 48 hours, the proportion of positive cells was observed under fluorescence microscope and cells were passaged after 90% confluence. Positive reporter cells are sorted by flow cytometry and expanded as stable cell lines for high-content screening analysis.

### Method details

#### Fabrication of the 3D micro-chip

The polymethyl methacrylate (PMMA) 3D micro-chip template containing 12 X 4 1 mm diameter wells with 4.5 mm center-to-center spacing were laser-engraved on 0.5 mm-thick PMMA sheets using Rayject 50 Laser Engraver. Gelatin microscaffold array was fabricated by adapting a previously reported method (Yan et al., 2015). Briefly, 12.48 g gelatin extracted from pork skin was completely dissolved in 100 mL de-ionized H_2_O to a concentration of 12.48% w/v at 60°C. After treating the PMMA array by Plasma Cleaner (Mycro Technologies) for 2 min, the PMMA array filled with gelatin precursor solution underwent cryogelation for 2 h in a −20°C refrigerator, and then was lyophilized for 2 h (Boyikang). The 3D gelatin microscaffold array was UV-sterilized for 4 h before cell culture.

#### High content screening analysis

The hepatic fibrotic biomimetic micro-niche was screened out by high content screening. HepaRG-mCherry, LSEC-GFP and LX-2-YFP cell lines were digested into single cells and resuspended medium with type I collagen at a ratio of 2:2:1. The type I collagen (3.35 mg/ml) accounted for one-third of the total liquid volume. The micro-niche medium is a mixture of HepaRG, LX-2 and LSEC cell medium containing half volume of Williams’ E medium, half volume of DMEM medium, 1x GlutaMax, 5μg/ml Insulin, 0.1 nM dexamethasone, 12% FBS and 1% PS. The micro-niche suspension was plated on the 3D micro-chip at a density of 30,000 cells per well for a volume of 3 μl, and incubated at 37°C for around 40 min. After matrix gelation, micro-niche medium was added to the plate and replaced daily. The three types of cells self-organized into 3D micro-tissues when pre-cultured in the microchip for 5 days. The pre-cultured micro-tissues were treated with 22 profibrotic factor combinations. The micro-tissues were stained with Hoechst 33342 after induction for 3 days, and then were imaged on Operetta using water objective lens and LED light source. Operation of high content imaging was performed according to the manufacturer’s recommendation (PerkinElmer).

#### Cell proliferation assay

CellTiter-Blue assay was used to examine the cell viability. Firstly, the calibration curve was plotted for the HepaRG-mCherry, LSEC-GFP, and LX-2-YFP mixed at 2:2:1 ratio with a series of total cell numbers. Calculation of cell proliferation within the different fibrotic liver micro-tissues was accomplished according to the calibration curve.

#### Niche extract production

To produce abundant NE, an amplified culture system based on 96-well round-bottomed ultra-low attachment plate (Corning) was adopted to replace the 3D microchip for fibrotic niche establishment which upgraded the total volume from 3 μl to 30 μl with no change in cell ratio and type I collagen concentration. The micro-niche suspension was plated at a density of 1.8×10ˆ5 cells per well in a volume of 30 μl. The amplified micro-tissues were collected into a 1.5 ml centrifuge tube and frozen at 80°C. Before producing the niche-derived matrix, the microtissue samples were thawed on ice, and 15 μl of 1 mg/ml DNase was added to each well. The thawed samples were grounded by an electric tissue grinder until there were no visible particles. Pepsin (10 mg/ml) was added to each tube to reach a final concentration of 1mg/ml and placed at 4°C for 48 h to obtain a uniform and stable NE. NE was aliquoted and stored frozen at −80°C. All enzyme solutions were prepared in PBS buffer, and sterilized by 0.22 μm filter membrane before use. The total protein concentrations were measured using the enhanced BCA Protein Assay Kit (Beyotime® Biotechnology, China).

NE-coated well plates were used to culture cells. First, we adjusted the pH of the NE solution to neutral with 1M NaOH. Then the 96-well plates were coated with NE at a protein density of 100 μg/cm^2^, which spontaneously formed a gel, and were air-dried in the safety cabinet. Matrigel with the same protein concentration was suspended in PBS as a control.

To prepare for the defined ECM resolved from NE, collagen type I (BD), type III (Sigma) and type IV (Sigma) with the original concentration of 2 mg/ml were diluted with deionized water at a ratio of 1: 5. 96-well plates were coated with these matrices at a density of 50 μl per well. The inflammatory factors IL-17, IL-18 and M-CSF were added to hepatoblasts expansion medium at a final concentration of 40 ng/ml.

#### NE imaging by scanning electron microscopy

The spontaneously formed NE gel was fixed with 2.5% glutaraldehyde in 1× PBS for 2 h at room temperature. The fixed samples were washed three times with PBS and dehydrated with a series of ethanol solutions of increasing concentration, 70%, 80%, 90%, and 100% and twice 100% with each step lasting 10 min. We removed as much ethanol as possible from the sample and added tert-butanol that just covered the sample. The sample was stored in tert-butanol at 4°C until further use. Freeze drying of samples was performed by using critical point drying (Tousimis Samdri-795). The dried samples were covered with a 30 nm layer of Au using a Quorum Q150T ES sputter. The electron microscopy analysis was performed using a Quanta 200 Scanning Electron Microscope, operating with an acceleration voltage of 5 keV and working distance of 5 mm.

#### RNA isolation and quantitative RT-PCR

TRIzol RNA isolation reagents (Invitrogen) were used to isolate the RNA in each group. Particularly, 3D microtissues and 3D organoids were pipetted thoroughly and treated with total RNA extraction reagent for 10 min. PCR with a HiScript II qRT SuperMix Kit (V) (Vazyme) was used to reverse transcript 500 ng of RNA into cDNA. Levels of gene expression were measured with real-time PCR using AceQ qPCR SYBR green master mix (Vazyme), and CFX96 machine (Bio-Rad). Relative gene expression was quantified using the 2ˆΔΔCt method and internally normalized to the expression of housekeeping gene GADPH. The primers are listed in Table S3.

#### Immunofluorescence staining and organoids visualization after tissue clearing

Cells were fixed with 4% PFA for 15 min and were washed three times by PBS, which were permeabilized with PBS containing 0.5% Triton X-100 (Sigma) for 10 min and blocked with PBS containing 5% BSA (Amresco) for 1 h. Next, cells were incubated overnight at 4°C with primary antibodies diluted in blocking solution. After washing, cells were incubated with the corresponding secondary antibodies if necessary. Cell nuclei were stained by Hoechst 33,342 (Beyotime, 1:2000) for 20 min. ImageJ and Imaris were used for image quantification.

For 3D liver organoids, Visikol HISTO-M Starter kit (Life Technologies) was used for tissue clearing to facilitate visualization. The organoids were fixed with 4% PFA for 30 min, and washed three times in PBS. The fixed organoids were dehydrated with first washing with 50% methanol in PBS, second washing with 80% methanol in deionized water, and third washing with 100% methanol. After that, the dehydrated organoids were rehydrated by first washing with 20% DMSO in methanol, second washing with 80% methanol in deionized water, third washing with 50% methanol in PBS, fourth washing with 100% PBS, and fifth washing with 0.2% Triton™ X-100 in PBS.

Samples were incubated in Visikol HISTO Penetration Buffer (PBS with 0.2% Triton™ X-100, 0.3 M glycine, and 20% DMSO; store at 2–8°C) with gentle shaking for about an hour. The samples were blocked in Visikol HISTO Blocking Buffer (PBS with 0.2% Triton™ X-100, 6% fetal bovine serum, and 10% DMSO; store at 2–8°C) with gentle shaking at room temperature for about an hour. The samples were transferred to primary antibody dilutions prepared in Visikol HISTO Antibody Buffer (PBS with 0.2% Tween™ 20, 10 μg/mL heparin, 3% fetal bovine serum, and 5% DMSO; store at 2–8°C) and incubated at 4°C with gentle shaking overnight. Following that, the samples were washed for 5 times in Visikol HISTO Washing Buffer (PBS with 0.2% Tween™ 20 and 10 μg/mL heparin; store at 2–8°C) with gentle shaking and incubated in secondary antibody dilutions in Visikol HISTO Antibody Buffer at RT with gentle shaking for about three hours. In Visikol Wash Buffer with gentle shaking, the samples were washed 5 times then incubated with Hoechst 33342 to a dilution of 1:1000 at RT about half an hour. First, using 50% ethanol in PBS the organoids started to dehydrate, then with 80% ethanol in deionized water, and finally in 100% ethanol with gentle shaking. Finally, we removed as much ethanol as possible from the sample, added Visikol HISTO-M, and incubated for 15 min. The cleared samples were imaged by a Nikon A1Rsi inverted confocal microscope.

#### Flow cytometry analysis

To obtain suspended cells, 2D cultured cells were digested with trypsin-EDTA and cells cultured in 3D scaffold were digested with PBS containing 0.4% Collagenase I (Life Technologies) and Accutase at 37°C for 40 min. The suspended cells were treated with the following reagents: 4% paraformaldehyde for 15 min; PBS containing 0.5% Triton X-100 for 10 min; PBS containing 5% BSA at room temperature for 1 h; PBS containing 5% BSA and the first antibodies at 37°C for 1 h; PBS containing the second antibodies at 37°C for 1 h. Finally, over 10^4^ immune-stained cells were analyzed using Flow cytometer LSRFortessa SORP (BD). The scaffolds would degrade and allow the collection of cells inside the scaffolds for the above process.

#### EdU staining

Hepatoblasts proliferation was measured by using the BeyoClick™ EdU Cell Proliferation Kit with Alexa Fluor 488 (Beyotime® Biotechnology, China, C0071S). According to the manufacturer’s recommendations, cells were treated with medium containing 10 μM EdU for 3 h to label proliferating cells. After incubation, cells were washed three times with Knockout DMEM basic medium and fixed in 4% PFA before permeabilization with PBS containing 0.5% Triton X-100 for 15 min. Next, the cells were treated with Click Reaction Buffer and were incubated at room temperature in the dark for 30 min. After washing, cell nuclei were stained with Hoechst 33342 (1:2000) for 20 min. Stained samples were observed under a Nikon A1Rsi inverted confocal microscope.

#### Endothelial network formation

hESC-derived ECs (55,000 viable cells/cm^2^) were seeded on a 24-well polystyrene plate coated with Ctrl-NE, Matrigel, P2-NE and F7-NE using AATS medium with 50 ng/ml VEGF (Peprotech) plus 20 ng/ml bFGF (Peprotech), and incubated at 37°C, 5% CO_2_. At 16 h after seeding, phase contrast images of tube formation were collected for angiogenesis analysis. The morphometric analysis was performed by Image J Angiogenesis Analyzer, with an output of parameters including nodes, segments, meshes, and network length.

#### mRNA sequencing and analysis

Total RNA of microtissues and liver organoids were isolated using the TRIzol RNA isolation reagents and 5 μg of total RNA were shipped on dry ice to Anoroad Gene Technology Corporation (Beijing, China) for RNA sequencing. Briefly, RNA purity was checked using the kaiaoK5500® Spectrophotometer (Kaiao, Beijing, China), RNA integrity and concentration were assessed using the RNA Nano 6000 Assay Kit of the Bioanalyzer 2100 system (Agilent Technologies, CA, USA). Samples were not sequenced until their quality was acceptable. Sequencing libraries were generated using NEBNext® Ultra™ RNA Library Prep Kit for Illumina® (#E7530L, NEB, USA) following the manufacturer’s recommendations and index codes were added to attribute sequences to each sample. The low-quality reads and adaptor sequences were trimmed with Trimmomatic. Clean reads were aligned to human genome by HISAT2. Gene counts were calculated by counting the overlap of reads on each gene with HT-seq, and the expression level was normalized as RPKM with gene annotation file from Ensembl and edgeR package in R. Differential expression genes were identified by DESeq2 package, and functional enrichment for Gene Ontology (GO) and KEGG were performed with GOstats package.

#### Quantitative proteomics

Both sample processing and LC−MS/MS (UltiMate 3000 RSLCnano and Q Exactive from Thermo Fisher) analysis were completed by the protein platform of the Tsinghua University Protein Research Technology Center. The sample processing method was based on the article published by Prof. Deng Haiteng’s Laboratory of Tsinghua University (Gu et al., 2015). Briefly, micro-tissue was treated with a protein extract buffer composed of 8 M urea and 1% protease inhibitor. 100 μg of peptides were eluted by incubation with DTT, and alkylated with iodoacetamide at room temperature in the dark. The enriched peptide samples were digested with trypsin at room temperature for 20 h. The peptides after trypsin digestion were desalted and labeled with TMT reagent (Thermo, Pierce Biotechnology, Rockford, IL).

The peptides were separated on a reverse phase column (0.075×150 mm inner diameter) packed with 1.8 μm C18 particles (Dr. Maisch GmbH, Ammerbuch Entringen, Germany) using a 120 min gradient elution at a flow rate of 0.25 μl/min. The elution buffer contains mobile phase A (0.1% formic acid in water) and mobile phase B (0.1% formic acid in acetonitrile). The liquid chromatography was coupled to the LTQ Orbitrap (Thermo Fisher Scientific, Germany) via a Proxeon nano-electrospray ionization source. The mass spectrometer was operated in data-dependent mode with Xcalibur software (version 3.0) and full scan mass spectrum was acquired, followed by 20 data-dependent MS2 scans at higher energy collisional-based fragmentation. Original data from MS/MS spectra of each LC-MS/MS run were analyzed using the SEQUEST search engine from Proteome Discoverer Software (version 2.1, Thermo-Fisher Scientific, United States) against the UniProt human database.

#### 3D liver organoids formation

To construct 3D liver organoids *in vitro*, H9 ESCs-derived hepatoblasts, H1 ESCs-derived ECPs, and human MSCs (inactivated with 30 μg/ml mitomycin treatment for 2 hours) were digested into single cells and resuspended in organoids growth medium with fibrotic matrix at a ratio of 5:5:1. The fibrotic matrix, which accounts for one-third of the total liquid volume, is composed of NE matrix (3.35mg/ml) and type I collagen (3.35mg/ml) in a 3: 1 ratio. For the Matrigel group, the volume of Matrigel corresponded to the same amount of protein as NE, other procedures were the same as NE groups. Organoids growth medium is a mixture of endothelial cells differentiation medium and hepatoblasts expansion medium which contains half volume of AATS medium, half volume of DMEM/F12 medium supplemented with 50 ng/ml VEGF, 10 ng/ml bFGF, 10 ng/ml nicotinamide, 1x Glutamax, 40 ng/ml HGF, 20 ng/ml EGF, 1x insulin, 1x transferrin, 1x selenium, 0.1 nM dexamethasone, 10% FBS and 1% PS. The organoid suspension was plated onto a 96-well round-bottom ultra-low attachment plate (Corning) at a density of 30,000 cells per well in a volume of 3 μl, and incubated at 37°C for around 40 min. After matrix gelation, organoid growth medium was added and replaced daily. Cultured organoids were maintained at 37°C in an atmosphere of 5% CO2, 95% air.

#### Atomic force microscopy for Young’s modulus testing

The stiffness of 3D organoids was measured with atomic force microscopy (AFM). After removing an organoid from the ultra-low attachment plate, one-third of the organoid volume was embedded in Matrigel, which was constructed on sterilized 25 mm-diameter coverslips and incubated at 37°C for 40 min. AFM was performed using the AFM module of CellHesion200 (JPK Instruments, Germany) that is mounted on an inverted optical microscope (Zeiss Observer A1 stand). The AFM probes consisted of a Tipless silicon sensor (ARROW-TL1-50, NANOWORLD) with a modified AFM cantilever which had a nominal spring constant of 0.03 N/m. The cantilever tip was attached with a plain microsphere (20 μm in diameter) to indent the organoids for the purpose of simplifying the contact geometry and minimizing the lateral strain of the sample during indentation. Before measuring stiffness of organoids, the cantilever was first calibrated on the glass coverslip using the thermal vibration method, where the resulting thermal spectrum was fitted with Lorentzian function to determine the spring constant. Individual organoid was indented approximately at the center of the organoid spheres under a piezo-actuated displacement rate of 5 μm/s. The Young’s modulus of each organoid was obtained by analyzing the force versus indentation curves using JPKSPM Data Processing software with the classical Hertz model. The minimum number of indentation locations at the center of each organoid was 20 times to ensure accurate measurement. All AFM measurements were performed in PBS at 37°C and results were obtained from triplicate samples per experimental condition.

#### Urea assays for hESC-derived hepatocyte-like cells

After exposure of the cells to 2 mM ammonium chloride (Sigma-Aldrich) for 24 h, urea productions in the culture media of hepatocyte-like cells were measured using Urea Assay Kit (BioAssay Systems). Fresh culture medium supplemented with 2 mM ammonium chloride was used as a negative control. Operation was performed according to the manufacturer’s recommendation.

### Quantification and statistical analysis

Statistical analyses were performed using one-way or two-way analysis of variance (ANOVA) with the GraphPad Prism 7.0. Multiple comparison between the groups was performed using post hoc Tukey’s multiple comparisons test (for one-way ANOVA) and Sidak’s multiple comparisons test (for two-way ANOVA). For experiments comparing two groups, we performed a two-tailed unpaired Student’s t-test. All results were obtained from at least triplicate independent samples unless otherwise stated. Sample sizes (n) are shown in captions and original data are shown in source data file. Statistical differences were represented by p value, which ns represents not statistically significant, ∗ represents p value smaller than 0.05, ∗∗ represents p value smaller than 0.01, ∗∗∗ represents p value smaller than 0.001, ∗∗∗∗ represents p value smaller than 0.0001. (Exact p values are presented in the figure legends). Data were expressed as means ± SEM.

## Data Availability

• Microscopy data reported in this paper will be shared by the lead contact upon request. • This paper does not report original code. • Any additional information required to reanalyze the data reported in this paper is available from the lead contact upon request.
