# Synthetic liver fibrotic niche extracts achieve *in vitro* hepatoblasts phenotype enhancement and expansion

**DOI:** 10.1016/j.isci.2025.113010

**Published:** 2025-07-02

**Authors:** Yuying Zhang, Anqi Guo, Cheng Lyu, Ran Bi, Zhaozhao Wu, Wenjing Li, Peng Zhao, Yudi Niu, Jie Na, Jianzhong Jeff Xi, Yanan Du

## Main text

(iScience *24*, 103303; November 19, 2021)

In the initial publication of this article, three errors were identified in the preparation and assembly of figures.

• In Figure 4D, the images representing the “D5 Ctrl-NE” and “F7-NE” conditions were inadvertently duplicated during figure assembly. This error has now been rectified by providing the corrected “D5 Ctrl-NE” image below.
• In Figure S2E, the image showing hESC-derived hepatoblasts cultured under Matrigel conditions was identical to an image previously published by the same authors (Figure 6B [bright image of 40kPa] in Guo et al., 2020, Cell Regen. *9*, 15, https://doi.org/10.1186/s13619-020-00054-4). Upon reviewing the original data from both papers, we determined that these experiments involving hESC-derived hepatoblasts cultured on various substrates with Matrigel coating were conducted concurrently by the same authors. An inadvertent image duplication occurred during figure assembly. The corrected Figure S2E is shown below.
• In Figure S6D, the images corresponding to the “Col III+IV+IL17” and “Col III+IL17+MCSF” conditions were inadvertently duplicated. This error has been corrected by incorporating the correct image for the “Col III+IV+IL17” condition from the original dataset, and the correct figure is shown below.

The authors have reviewed and verified all corrections and confirm that these errors do not alter the quantitative statistical results of the experiment and the scientific conclusions of the study. The authors feel regret of these oversights and sincerely apologize for any confusion or inconvenience caused to the readers.

**Figure:**
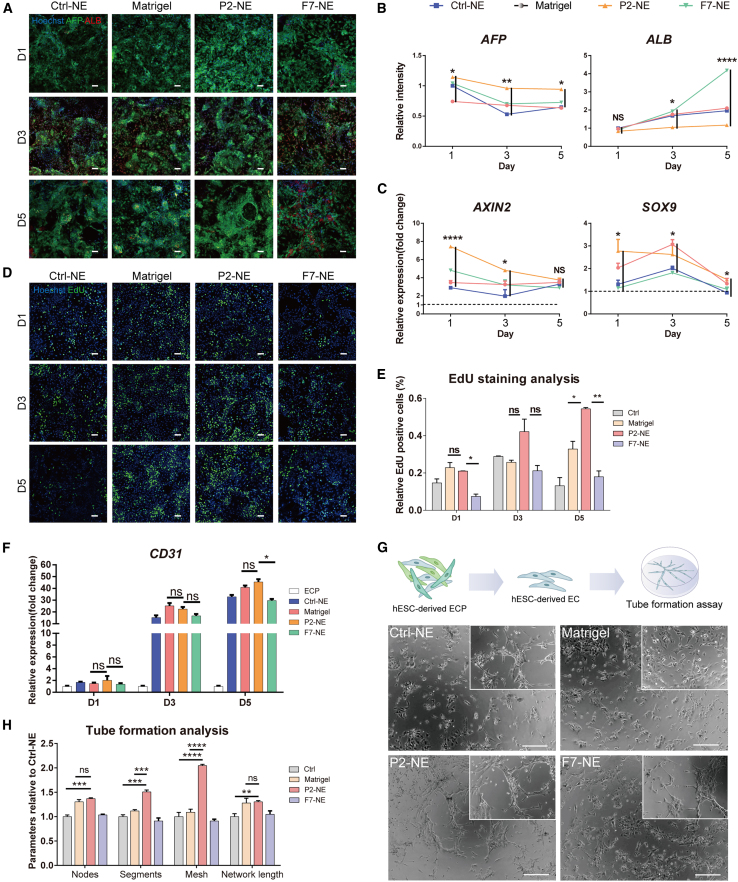
Figure 4. Synthetic fibrotic NE promoted phenotype maintenance of hESC-derived HBs and angiogenesis of hESC-derived ECs in 2D culture (corrected)

**Figure:**
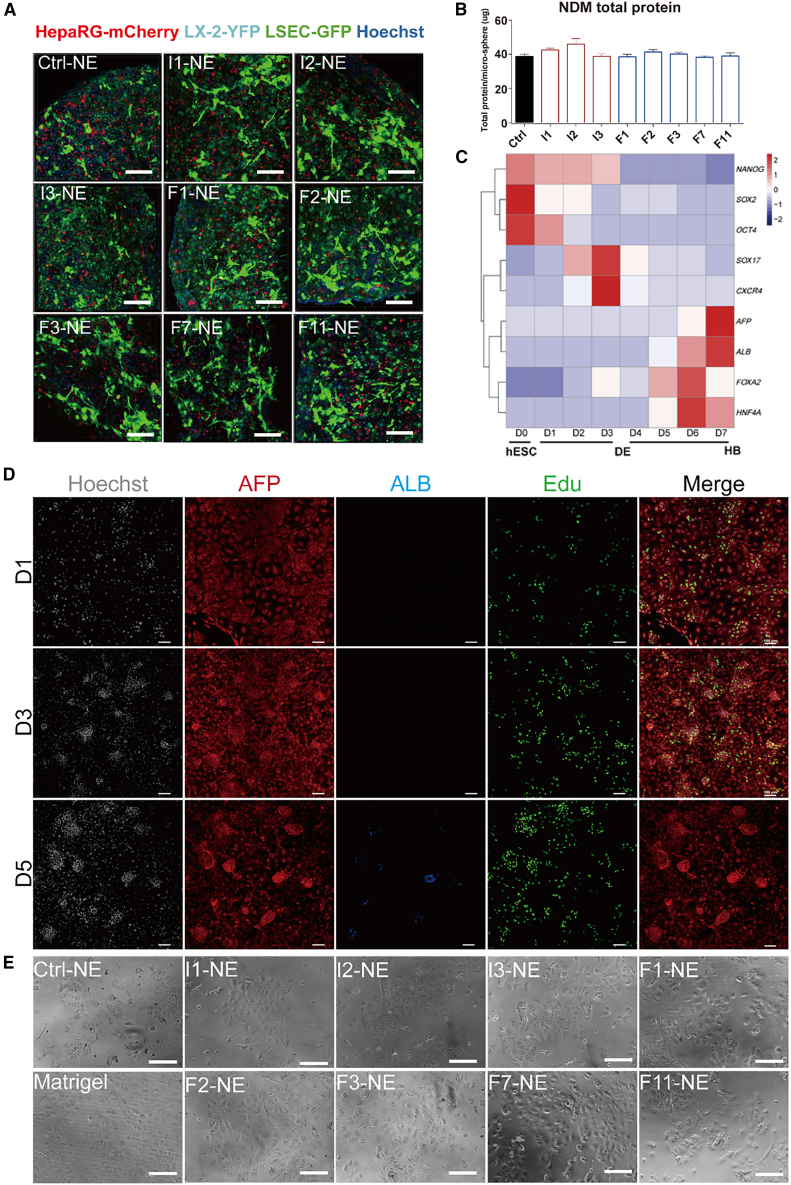
Figure S2. Phenotype maintenance of hESC-derived HBs on pre-selected fibrotic NE, related to Figure 2 (corrected)

**Figure:**
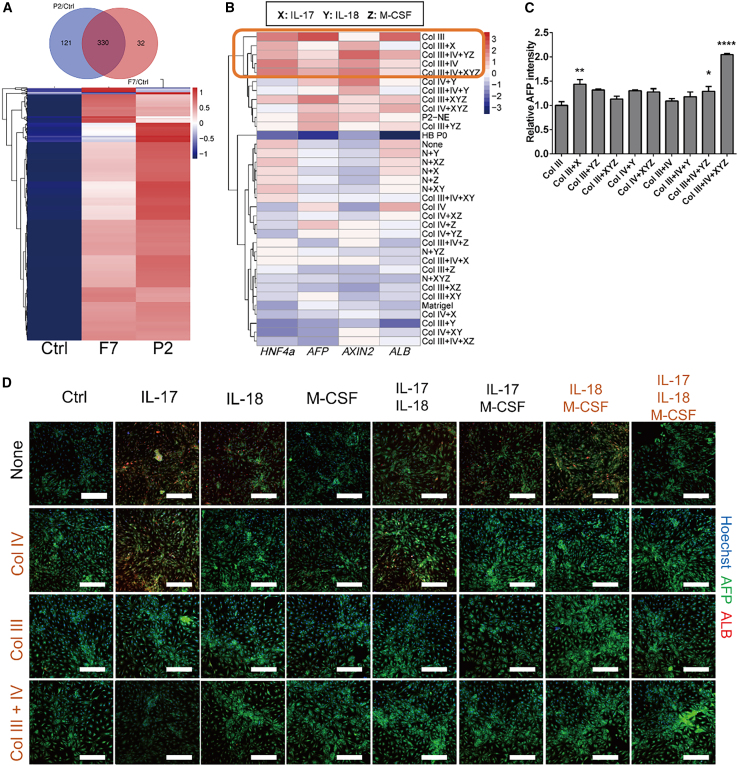
Figure S6. Differences in HBs phenotype maintenance and expansion with different defined ECM and factor combinations, related to Figure 6 (corrected)

