# Supplementary material for: Synthetic liver fibrotic niche extracts achieve in vitro hepatoblasts phenotype enhancement and expansion
Source: iScience. 2021 Oct 19;24(11):103303. doi: 10.1016/j.isci.2021.103303 (PMC8571728; doi:10.1016/j.isci.2021.103303)
Supplement: Document S1. Tables S1–S3 and Figures S1–S10 [file mmc1.pdf]

iScience, Volume *24*

Supplemental information

Synthetic liver fibrotic niche extracts achieve *in vitro* hepatoblasts phenotype enhancement and expansion

Yuying Zhang, Anqi Guo, Cheng Lyu, Ran Bi, Zhaozhao Wu, Wenjing Li, Peng Zhao, Yudi Niu, Jie Na, Jianzhong Jeff Xi, and Yanan Du

Table S1. 50 combinations of pro-fibrotic factors for synthetic liver fibrotic niches, related to Figure 3.

	APAP (mM)	TGFβ1 (ng/mL)	VEGF (ng/mL)	TNFα (ng/mL)	IFNγ (ng/mL)
1	—	—	25	40	40
2	—	—	50	40	40
3	—	—	25	20	20
4	—	—	50	20	20
5	0.625	5	—	20	20
6	0.625	10	—	20	20
7	0.625	20	—	20	20
8	1.25	5	—	20	20
9	1.25	10	—	20	20
10	1.25	20	—	20	20
11	2.5	5	—	20	20
12	2.5	10	—	20	20
13	2.5	20	—	20	20
14	0.625	5	—	40	40
15	0.625	10	—	40	40
16	0.625	20	—	40	40
17	1.25	5	—	40	40
18	1.25	10	—	40	40
19	1.25	20	—	40	40
20	2.5	5	—	40	40
21	2.5	10	—	40	40
22	2.5	20	—	40	40
23	0.625	5	25	20	20
24	0.625	10	25	20	20
25	0.625	20	25	20	20
26	1.25	5	25	20	20

	APAP (mM)	TGFβ1 (ng/mL)	VEGF (ng/mL)	TNFα (ng/mL)	IFNγ (ng/mL)
27	1.25	10	25	20	20
28	1.25	20	25	20	20
29	2.5	5	25	20	20
30	2.5	10	25	20	20
31	2.5	20	25	20	20
32	0.625	5	25	40	40
33	0.625	10	25	40	40
34	0.625	20	25	40	40
35	1.25	5	25	40	40
36	1.25	10	25	40	40
37	1.25	20	25	40	40
38	2.5	5	25	40	40
39	2.5	10	25	40	40
40	2.5	20	25	40	40
41	0.625	5	50	40	40
42	0.625	10	50	40	40
43	0.625	20	50	40	40
44	1.25	5	50	40	40
45	1.25	10	50	40	40
46	1.25	20	50	40	40
47	2.5	5	50	40	40
48	2.5	10	50	40	40
49	2.5	20	50	40	40

Table S2. Defined extracellular components within synthetic liver fibrotic niches analyzed by quantitative proteomics, related to Figure 6.

Accession	Description	Exp. q-value	Unique peptides	P2 NE/F7 NE Ratio
P02461	Collagen alpha-1(III) chain	0.004	2	2.112
Q14116	Interleukin-18	0.001	2	1.335
P09603	Macrophage colony-stimulating factor 1	0.002	2	1.281
P08572	Collagen type IV	0.001	3	1.226
P34931	Heat shock 70 kDa protein 1-like	0	3	1.126
P02675	Fibrinogen beta chain	0.028	1	1.168
P20908	Collagen alpha-1(V) chain	0	8	1.087
P18065	Insulin-like growth factor-binding protein 2	0.001	4	1.075
P35555	Fibrillin-1	0.002	2	1.063
Q5TAT6	Collagen alpha-1(XIII) chain	0.015	1	1.044
Q6PCB0	von Willebrand factor A domain-containing protein 1	0.002	1	1.034
O15230	Laminin subunit alpha-5	0	12	1.027
P41221	Protein Wnt-5a	0.001	2	1.026
P98160	Basement membrane-specific heparan sulfate proteoglycan core protein	0	68	1.021
P55268	Laminin subunit beta-2	0	14	1.02
P02458	Collagen alpha-1(II)	0.001	3	1.005
Q9HCB6	Spondin-1	0.009	1	0.982
Q13751	Laminin subunit beta-3	0.001	3	0.969
P05452	Tetranectin	0	3	0.956

Table S3. These primers are used for quantitative RT-PCR, related to STAR Methods.

Gene	Forward primer	Reverse primer
GAPDH	GGCTGAGAACGGGAAGCTTGTCAT	CAGCCTTCTCCATGGTGGTGAAGA
NANOG	TTTGTGGGCCTGAAGAAAACT	AGGGCTGTCCTGAATAAGCAG
OCT4	CTTGAATCCCGAATGGAAAGGG	GTGTATATCCCAGGGTGATCCTC
CD31	CACCTGGCCCAGGAGTTTC	AGTACACAGCCTTGTTGCCATGT
SOX17	CTCCGGTGTGAATCTCCCC	CACGTCAGGATAGTTGCAGTAAT
FOXA2	GGAGCAGCTACTATGCAGAGC	CGTGTTCATGCCGTTCATCC
AFP	TGTACTGCAGAGATAAGTTTAGCTGAC	TCCTTGTAAGTGGCTTCTTGAAC
ALB	AATGTTGCCAAGCTGCTGA	CTTCCCTTCATCCCGAAGTT
RBP4	CCAGAAGCGCAGAAGATTG	TTTCTTTCTGATCTGCCATCG
ZO-1	ACCAGTAAGTCGTCCTGATCC	TCGGCCAAATCTTCTCACTCC
AXIN2	TTATGCTTTGCACTACGTCCCTCCA	CGCAACATGGTCAACCCTCAGAC
SOX9	ACACACAGCTCACTCGACCTTG	GGGAATTCTGGTTGGTCCTCT
HNF4a	AGCAACGGACAGATGTGTGA	TCAGACCCTGAGCCACCT
ACTA2	GTGTTGCCCCTGAAGAGCAT	GCTGGGACATTGAAAGTCTCA
CD32b	GGGATCATTGTGGCTGTG	ATTAGTGGGATTGGCTG
LYVE-1	TGCAGAATTATGGGGATCA	GGCTGTTTCAACTTGGTCCT
STAB2	AGTGGACTATGGACCTAGACCCAAC	AGTAAGCAGCCAAGGCAACAGC
E-Cadherin	TGAAGGTGACAGAGCCTCTGGAT	TGGGTGAATTCGGGCTTGTT

Fig S1. High content screening for pre-selected biomimetic liver fibrotic niches, related to Figure 1.

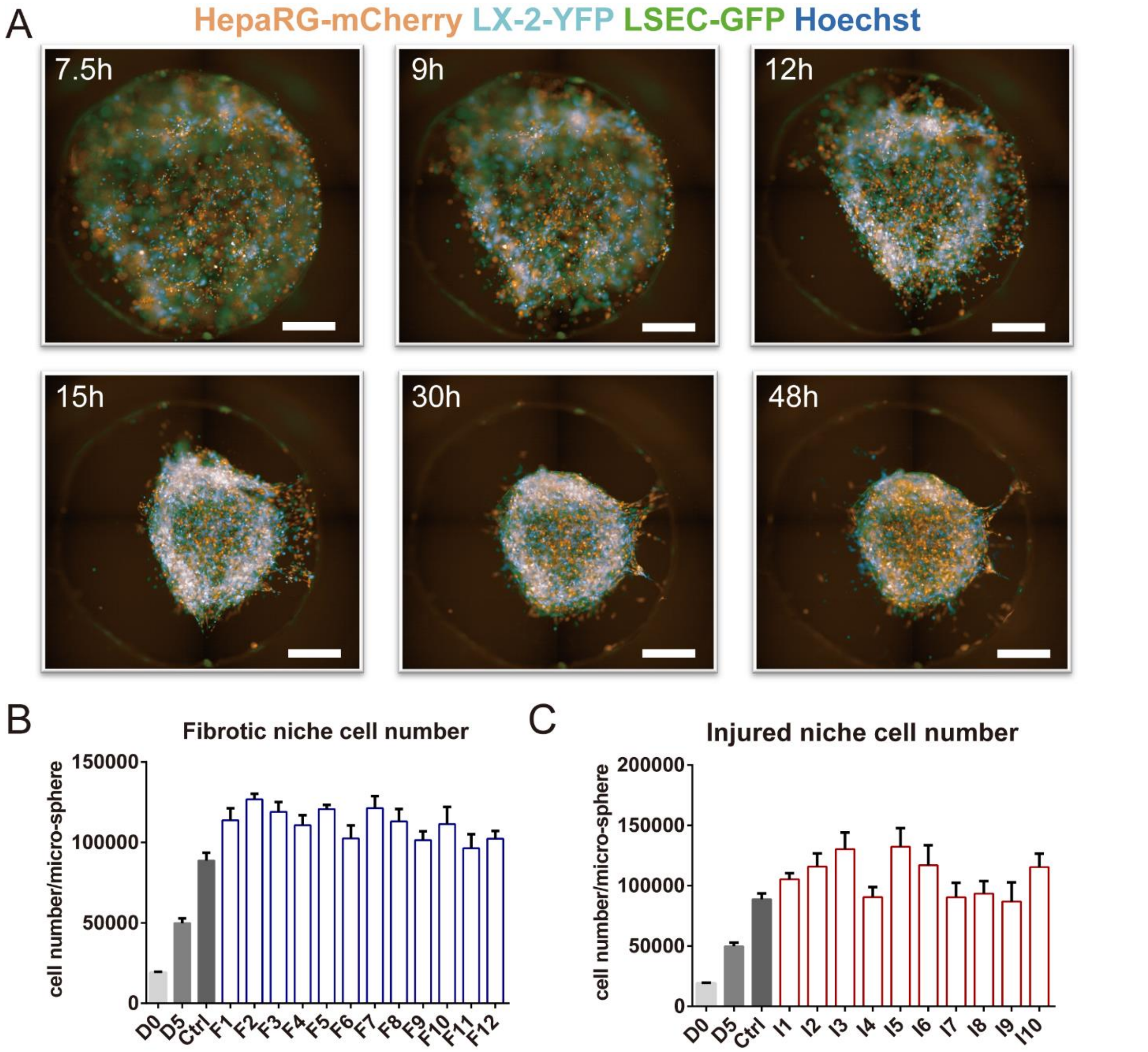

(A) Dynamic changes of 3D microtissue formed by the self-assembly of HepaRG-mCherry, LX-2-YFP and LSEC-GFP in a 3D microchip system. Scale bar, 400 μm.

(B,C) Changes in the total number of cells within the biomimetic liver injury niches and liver fibrosis niches; D0 is the initial number of cells plated per microtissue (1.8x10^4 cells); D5 is the number of cells per microtissue after 5 days' culture; I1-I10 and F1-F12 represent the number of cells within the pathological microtissues obtained after 3 days of factor/compound treatment on the basis of D5 microtissues, the control group (Ctrl) was not treated with any compound or factor.

Fig S2. Phenotype maintenance of hESC-derived HBs on pre-selected fibrotic NE, related to Figure 2.

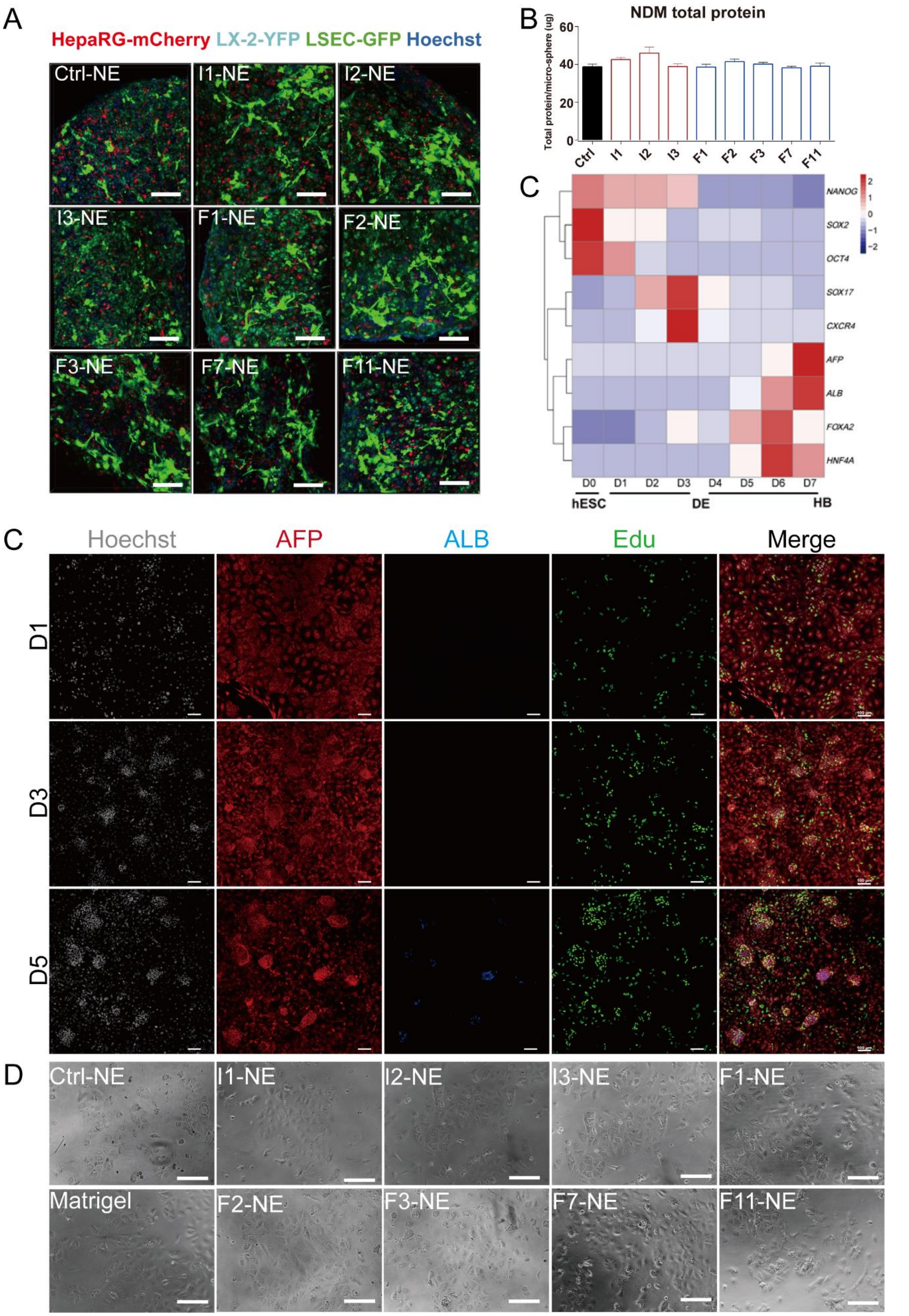

(A) The morphology of cells cultured in the pre-selected biomimetic liver fibrotic niches in 96-well ultra-low attachment plates. Cell morphology of HepaRG-mCherry, LX-2-YFP and LSEC-GFP within niches was photographed using laser confocal microscopy. Scale bar, 100 μm.
(B) Quantification of total protein amount within NE by BCA, shown as total protein amount within each microtissue cultured in 96-well plates.
(C) Changes in gene expression patterns over time during differentiation of hESC to definitive endoderm (DE) and HBs. The marker genes at hESC stage are *OCT4, NANOG* and *SOX2*, marker genes for DE stage are *SOX17* and *CXCR4*, marker genes for HB stage are *HNF4a* and *AFP*.
(D)AFP/ALB immunofluorescence and EdU staining of hESC-derived HBs cultured on plate coated with Matrigel on day 1, day 3 and day 5, with a scale bar of 100 μm.
(E) Cell morphology of hESC-derived HBs passaged onto different NE on day 1, with a scale length of 100 μm.

Fig S3. Quantification of matrix fiber thickness, related to Figure 2.

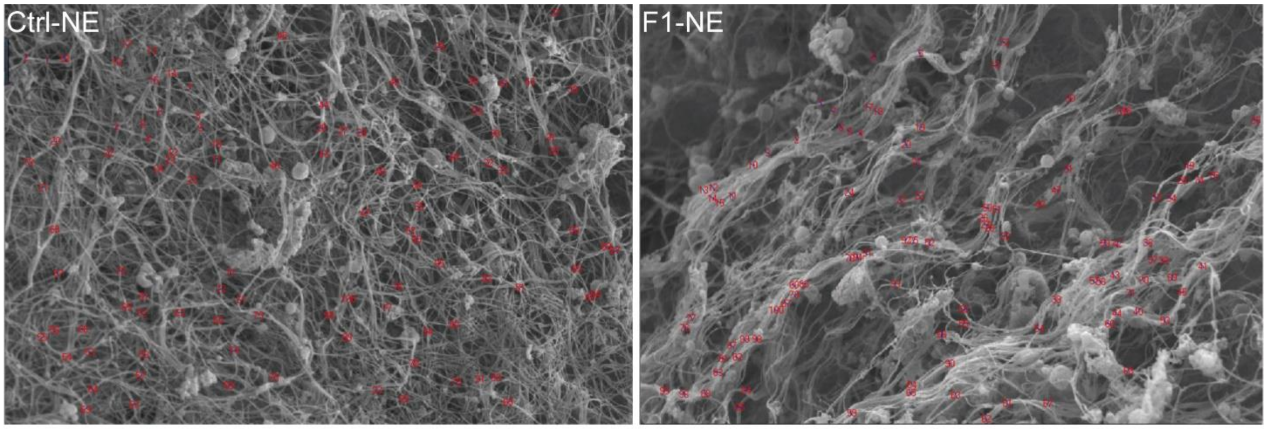

Max Size/μm	Minimal Size/μm	Average Size/μm
0.18	0.05	0.11

Max Size/μm	Minimal Size/μm	Average Size/μm
0.28	0.08	0.18

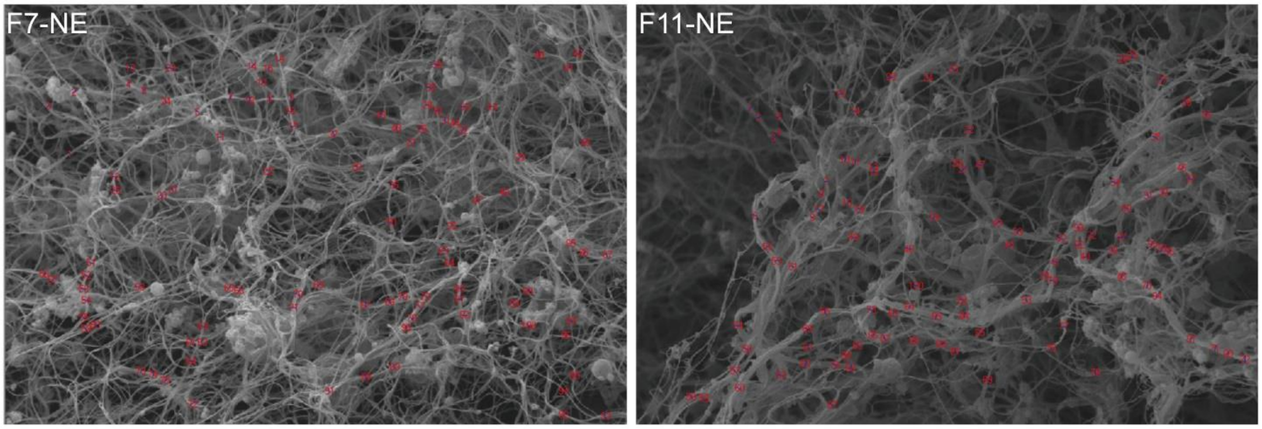

Max Size/μm	Minimal Size/μm	Average Size/μm
0.24	0.09	0.15

Max Size/μm	Minimal Size/μm	Average Size/μm
0.28	0.10	0.17

The SEM image was divided equally into four parts and opened in Nano Measurer. Scale size was defined using bar embedded in the raw image (1 μm). 25 objects in each dissected part (single fiber on focal plane) was randomly selected, and each object was marked with blue line to indicate length and red to indicate number. The thickness of each selected fiber was recorded. Then statistical analysis was performed for 100 points in each experimental group, including the removal of outliers, normality test and One-way ANOVA.

Fig S4. Characterization of optimized liver fibrotic niches, related to Figure 3.

A

	APAP (mM)	TGFβ1 (ng/mL)	VEGF (ng/mL)	TNFα (ng/mL)	IFNγ (ng/mL)
P1	0.625	5	25	20	20
P2	1.25	5	25	20	20
P3	2.5	5	25	20	20
P4	0.625	10	25	40	40
P5	2.5	10	25	40	40

B

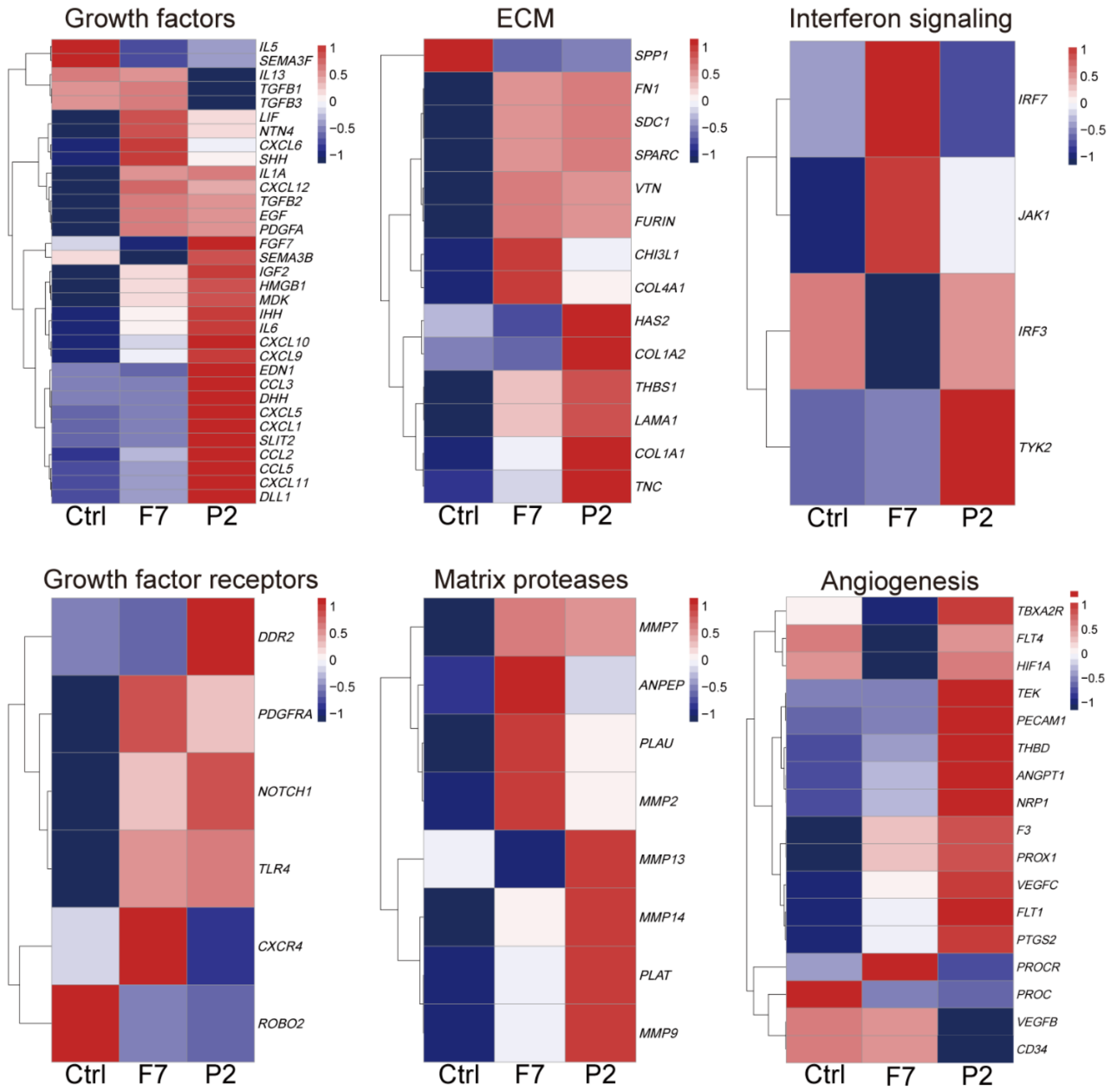

(A) 5 candidate profibrotic factor combinations for construction of the biomimetic fibrotic niches from a total of 50 conditions at Table 1.

(B) RNA sequencing analyzed the difference in gene expression between P2 niche and F7 niche. Hierarchical cluster analysis on the expression of key pathways involved in the fibrosis progression *in vivo*, namely growth factors, growth factor receptors, ECM, matrix proteases, interferon signaling pathways, and angiogenesis.

Fig S5. Gene expression of 3D organoids cultured *in vitro*, related to Figure 5.

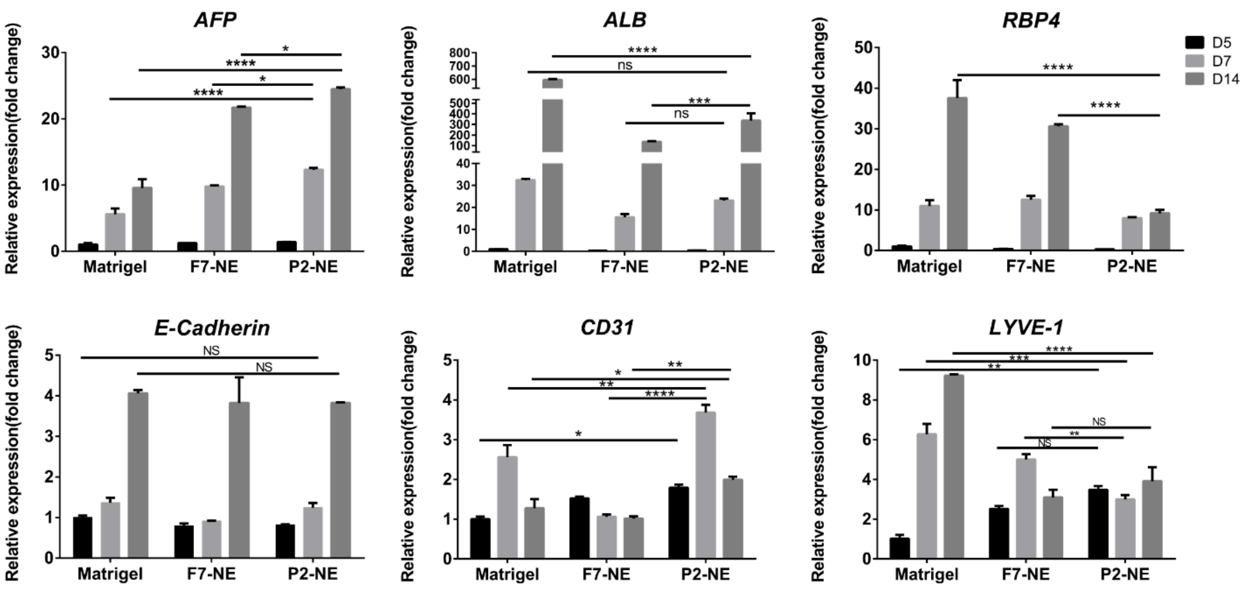

Matrigel, F7 NE, and P2 NE-assisted 3D organoids cultured *in vitro* at days 5, 7, and 14 were analyzed by qPCR for the expression of HBs marker genes *AFP*, HBs differentiation marker *ALB, RBP4* and E-cadherin, hESC-derived endothelial marker *CD31*, and the normal liver sinusoidal endothelial cell marker gene *LYVE-1*, *GAPDH* was used as housekeeping gene.

Fig S6. Differences in HBs phenotype maintenance and expansion with different defined ECM and factor combinations, related to Figure 6.

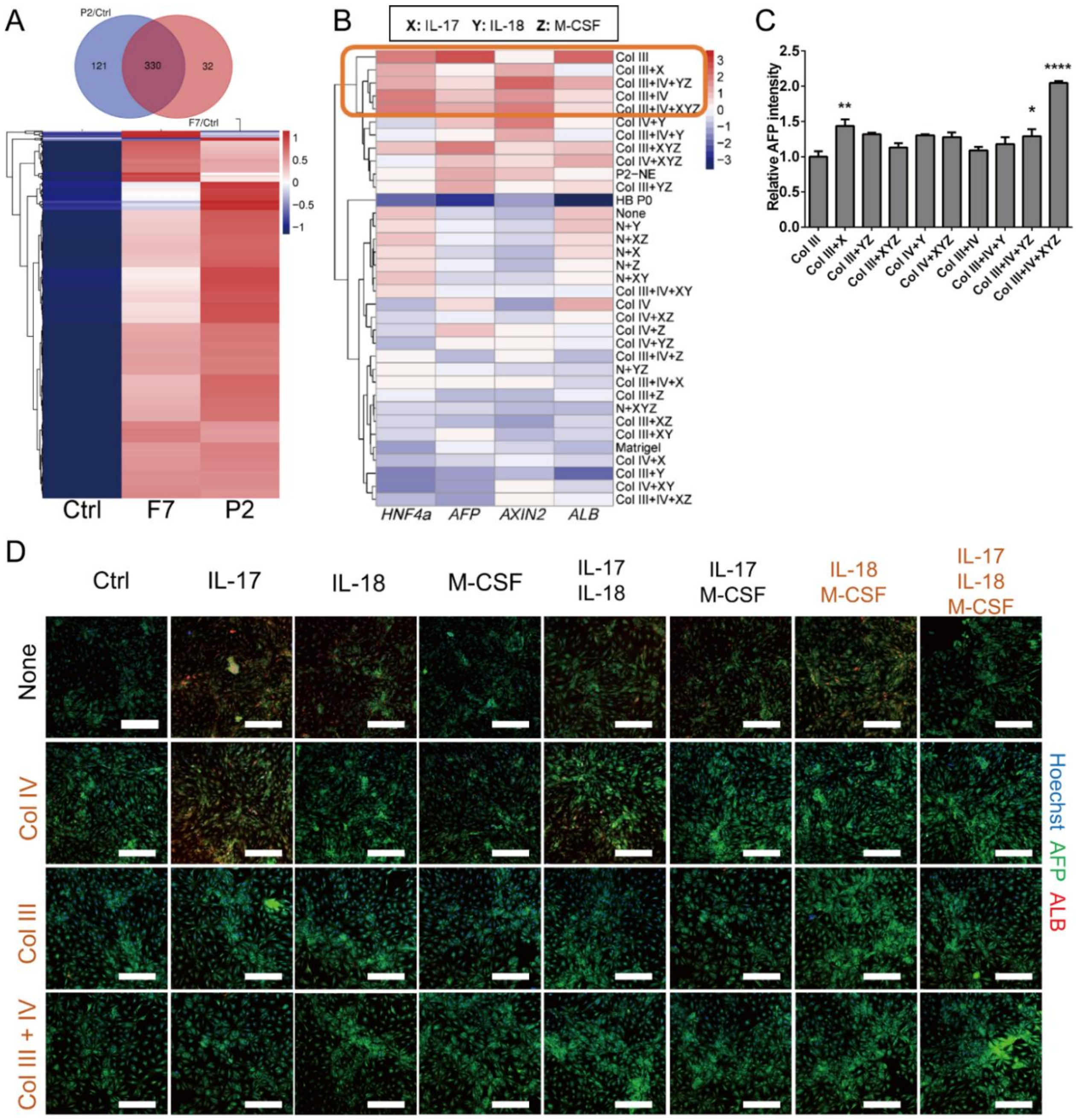

(A) Wayne plot analysis and z score standardized hierarchical cluster analysis for proteomic data generated from P2/Ctrl and F7/Ctrl;
(B) qPCR analyzed the expression of HBs marker genes *HNF4a*, *AFP*, and *AXIN2* and ALB for hESC-derived HBs cultured 3 days by different combinations of ECM and factors; the gene expression difference was demonstrated as a heat map.
(C, D) Immunofluorescence staining of AFP and ALB for hESC-derived HBs after 3 days *in vitro* culture by different combinations of ECM and factors, with scale bar of 200 μm;

Fig S7. The influence of Type I collagen combined with different ECM and factors on the HBs phenotype maintenance and expansion, related to Figure 6.

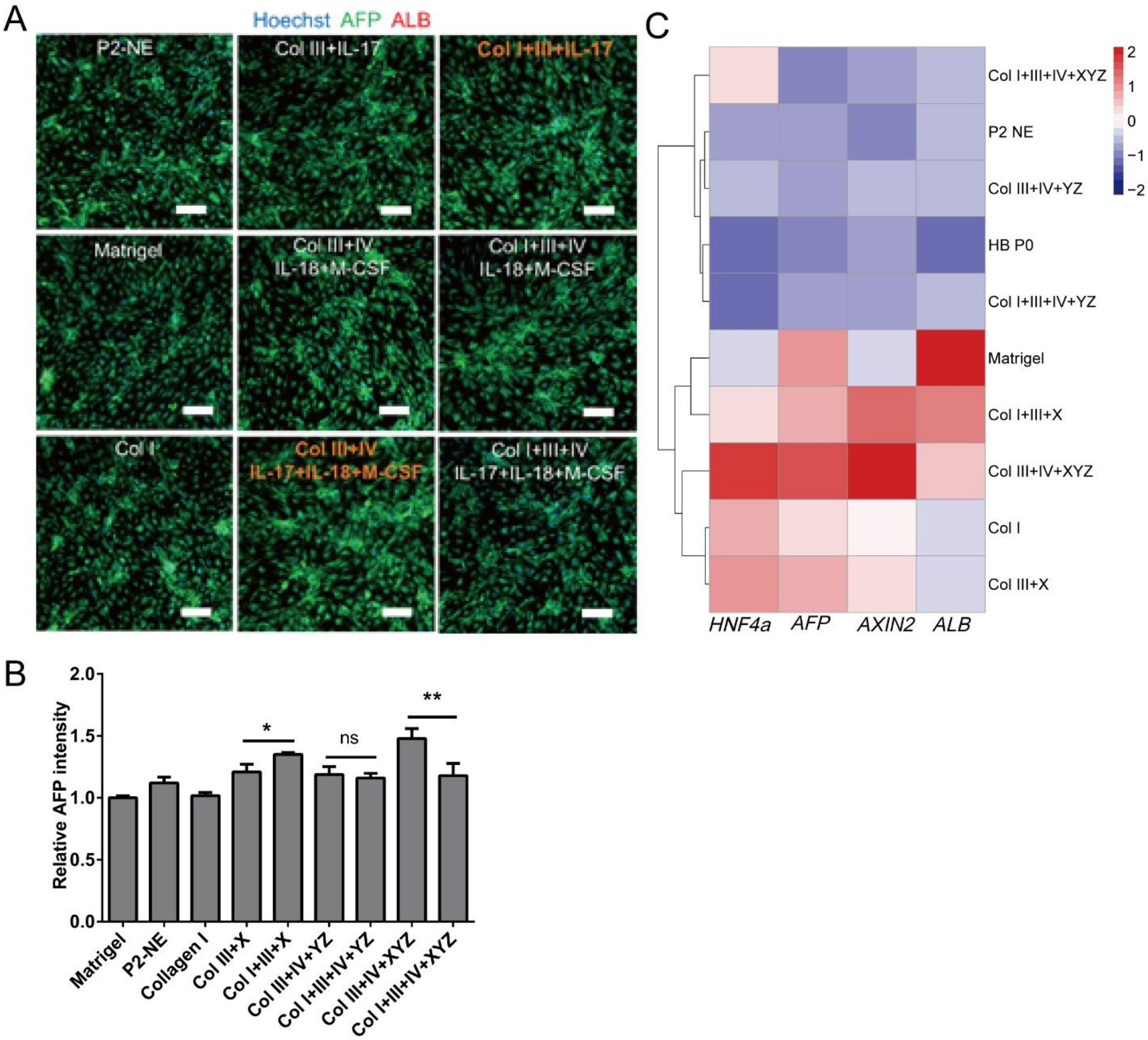

(A, B) AFP and ALB immunofluorescence staining of hESC-derived HBs after 3 days of *in vitro* culture by different ECM and factor combinations, scale bar: 100 μm;
(C) Gene expression clustering analysis on qPCR results for hESC-derived HBs after 3 days of *in vitro* culture by different ECM and factor combinations. X: IL-17, Y:IL-18, Z:M-CSF。HBs-R: HBs self-renewal.

Fig S8. qPCR show gene expression of HBs on different substrates for passages 1,2,3,5,7, which were passaged every 3 days, related to Figure 6.

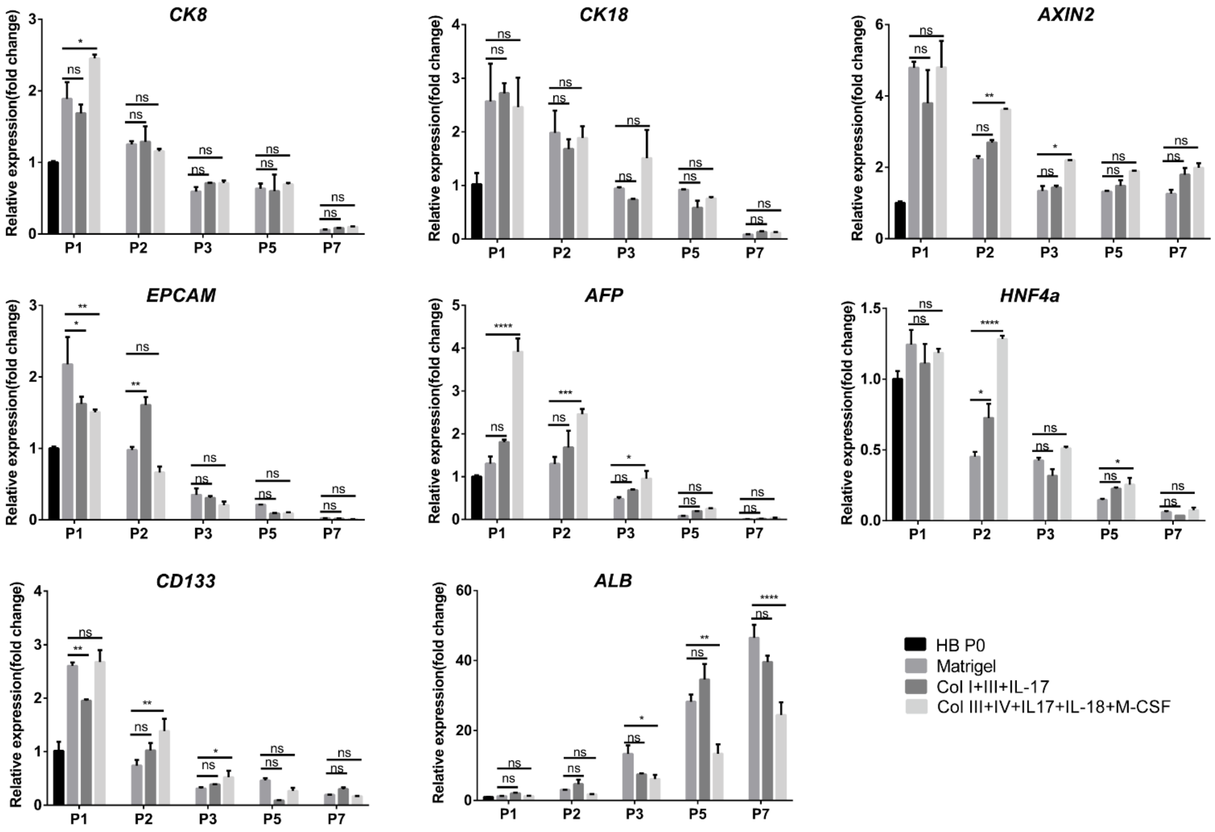

Fig S9. The potency of hepatic differentiation of HBs expanded with different combinations of ECM and factors, related to Figure 6.

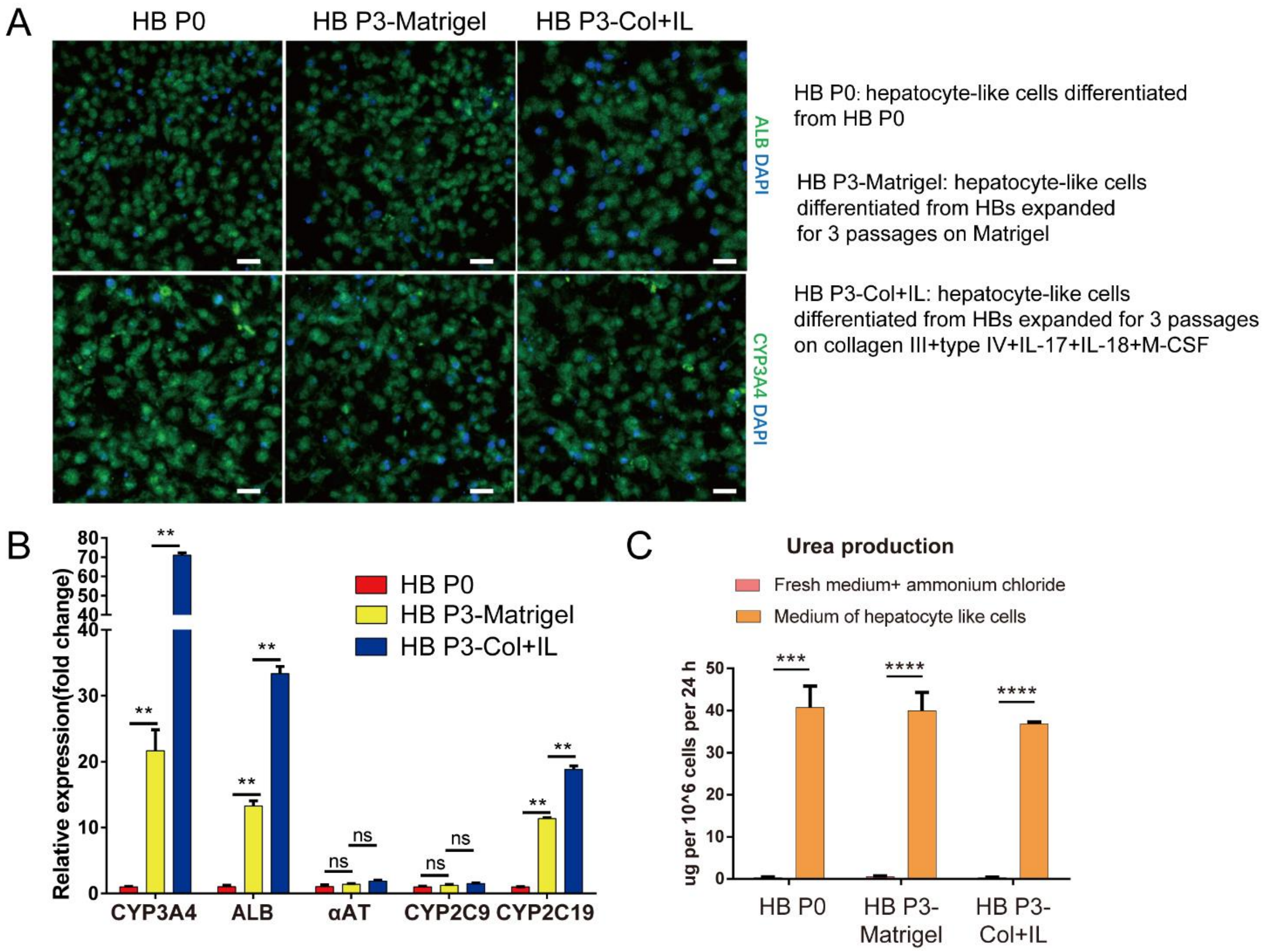

(A) Immunofluorescence staining of CYP3A4 and ALB for hepatocyte-like cells differentiated from HB P0 and HBs expanded for 3 passages on Matrigel and collagen III+ type IV+IL-17+IL-18+M-CSF respectively;
(B) qPCR show the expression the expression of mature hepatocyte marker genes CYP3A4, CYP2C9, CYP2C19, αAT and ALB for hepatocyte-like cells differentiated from HB P0 and 3rd passages of HBs expanded for 3 passages on Matrigel and under collagen III+ type IV+IL-17+IL-18+M-CSF respectively;
(C) Urea production for hepatocyte-like cells differentiated from HB P0 and 3rd passages of HBs expanded for 3 passages on Matrigel and under collagen III+ type IV+IL-17+IL-18+M-CSF respectively.

Fig S10. The influence of oxygen level combined with different ECM and factors on the HBs phenotype maintenance and expansion, related to Figure 6.

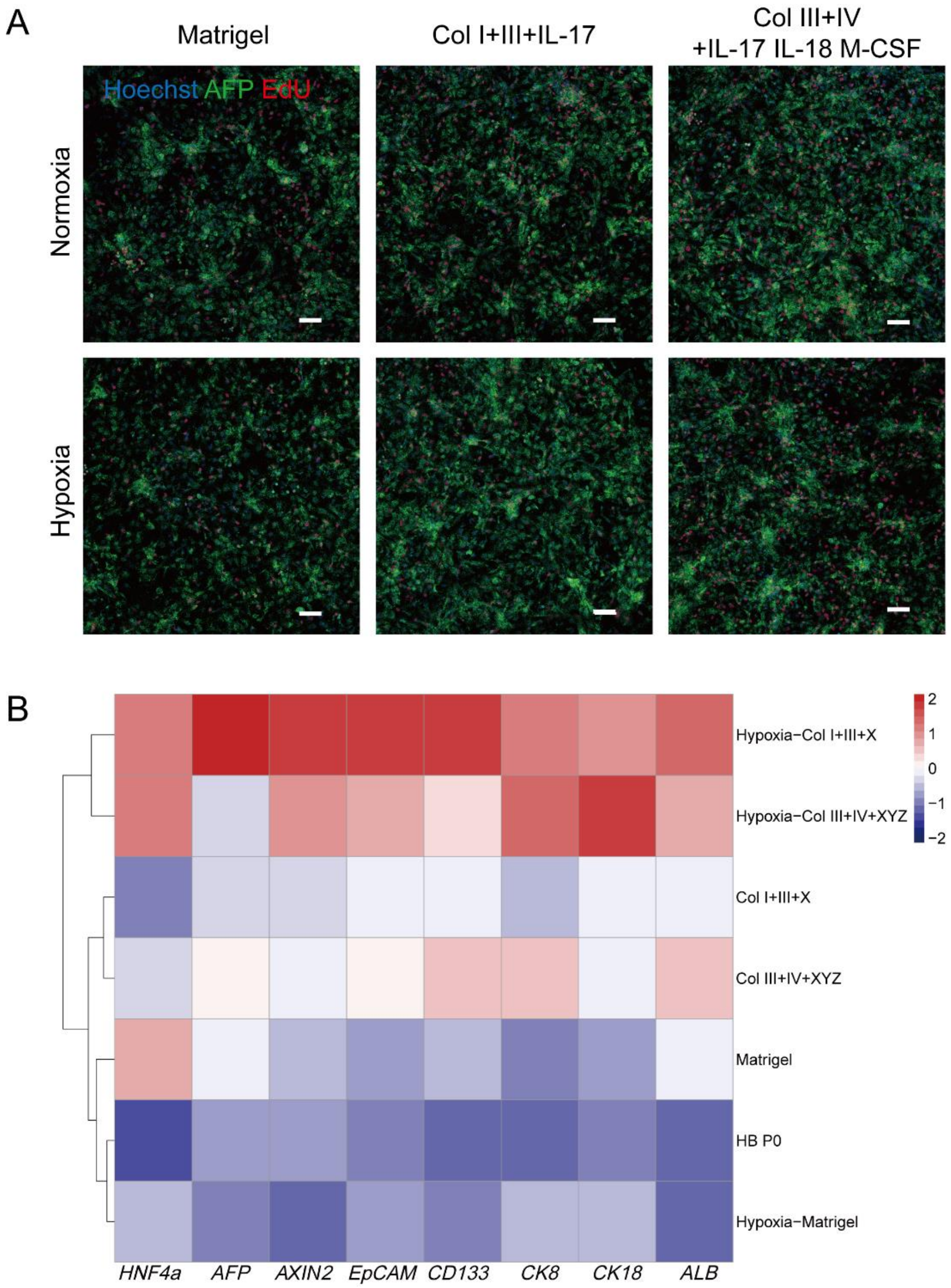

(A) Immunofluorescent staining of AFP and ALB in hESC-derived HBs after 3 days' culture under normal O2 level and hypoxia (5% O2) in two types of ECM and factors combination: Col I+III+IL-17 or Col III+IV+IL-17+IL-18+M-CSF. Scale length, 100 μm.

(B) Clustering analysis on qPCR results for the expression of HBs marker genes *HNF4a, AFP, AXIN2, EpCAM, CD133, CK8* and *CK18*, and *ALB* gene that represents HBs differentiation. X: IL-17, Y:IL-18, Z:M-CSF.
